# A dual role of YAP in driving TGFβ-mediated endothelial-to-mesenchymal transition

**DOI:** 10.1242/jcs.251371

**Published:** 2021-08-02

**Authors:** Cecilia Savorani, Matteo Malinverno, Roberta Seccia, Claudio Maderna, Monica Giannotta, Linda Terreran, Eleonora Mastrapasqua, Stefano Campaner, Elisabetta Dejana, Costanza Giampietro

**Affiliations:** 1 Institute of Molecular Oncology (IFOM), The Fondazione Italiana per la Ricerca sul Cancro (FIRC) Institute of Molecular Oncology, Milan 20139, Italy; 2 Center for Genomic Science of IIT@SEMM, Fondazione Istituto Italiano di Tecnologia (IIT), Milan 20139, Italy; 3 Department of Immunology, Genetics and Pathology, Vascular Biology, Uppsala University, Uppsala 751 85, Sweden; 4 Swiss Federal Laboratories for Materials Science and Technology (EMPA), Dübendorf 8600, Switzerland; 5 Department of Mechanical and Process Engineering, ETH Zurich, Zurich 8092, Switzerland

**Keywords:** YAP, Endothelial-to-mesenchymal transition, TGFβ pathway, SMAD3

## Abstract

Endothelial-to-mesenchymal transition (EndMT) is the biological process through which endothelial cells transdifferentiate into mesenchymal cells. During embryo development, EndMT regulates endocardial cushion formation via TGFβ/BMP signaling. In adults, EndMT is mainly activated during pathological conditions. Hence, it is necessary to characterize molecular regulators cooperating with TGFβ signaling in driving EndMT, to identify potential novel therapeutic targets to treat these pathologies. Here, we studied YAP, a transcriptional co-regulator involved in several biological processes, including epithelial-to-mesenchymal transition (EMT). As EndMT is the endothelial-specific form of EMT, and YAP (herein referring to YAP1) and TGFβ signaling cross-talk in other contexts, we hypothesized that YAP contributes to EndMT by modulating TGFβ signaling. We demonstrate that YAP is required to trigger TGFβ-induced EndMT response, specifically contributing to SMAD3-driven EndMT early gene transcription. We provide novel evidence that YAP acts as SMAD3 transcriptional co-factor and prevents GSK3β-mediated SMAD3 phosphorylation, thus protecting SMAD3 from degradation. YAP is therefore emerging as a possible candidate target to inhibit pathological TGFβ-induced EndMT at early stages.

## Abstract (precis)

**Summary:** A new crucial role for YAP as a co-activator of early pathological TGFβ-mediated endothelial-to-mesenchymal transition program and characterization of the underlying molecular mechanism.

## INTRODUCTION

Endothelial cells (EC), lining the inner surface of the vessel wall, are regulators of many physiological processes involved in the control of vascular homeostasis, such as the trafficking of cells and solutes between blood and underlying tissues, permeability, angiogenesis and immunity (Dejana, 2004). EC exhibit a significant heterogeneity in time, structure, exerted function, vascular location, health and disease (Aird, 2003, 2006). Interestingly, a growing amount of evidence demonstrates that EC are involved in the majority of human diseases, either as a cause or as a target of the damage induced by the disease (Rajendran et al., 2013). Owing to their remarkable plasticity, in pathological conditions, highly differentiated and specialized EC undergo endothelial-to-mesenchymal transition (EndMT), a process through which they transdifferentiate into mesenchymal-like cells and change their characteristics, acquiring mesenchymal features, such as spindle-shape morphology, proliferative, invasive and migratory properties, weakening of cell-cell junctions, loss of endothelial-specific markers, gain of mesenchymal markers and readjustment of the cytoskeletal organization (Lin et al., 2012; Dejana et al., 2017; Cho et al., 2018; Dejana and Lampugnani, 2018; Hong et al., 2018).

EndMT is a fundamental process during development. In the specific context of endocardial cushion morphogenesis, EC in the region of the developing atrioventricular canal undergo EndMT to originate mesenchymal cells forming the endocardial cushion tissue (Nakajima et al., 2000). In the adult, it has been widely demonstrated that EndMT plays a major role in chronic fibrosis-type injuries and diseases (Pardali et al., 2017). In the cardiovascular system, this includes contributions to the pathologies of vascular malformations (Maddaluno et al., 2013; Bravi et al., 2016), calcifications (Guihard et al., 2016), pulmonary hypertension (Zhang et al., 2018), myocardial infarction (Gong et al., 2017) and cardiac fibrosis (Zeisberg et al., 2007b; Sánchez-Duffhues et al., 2018). Moreover, it has been reported that EndMT generates cancer-associated fibroblasts, which are known to facilitate tumor progression (Potenta et al., 2008). All these findings suggest that targeting EndMT may be a novel therapeutic strategy, applicable not only to cancer but also to other diseases.

EndMT is regulated by a complex cross talk of several signaling pathways, among which TGFβ plays a pivotal role (van Meeteren and ten Dijke, 2012). However, the mechanisms of integration of these signals by cells are not fully understood. The intracellular effectors of TGFβ are the SMAD proteins that, activated by receptors, translocate into the nucleus, where they regulate gene expression (Heldin et al., 1997). Although this pathway is essentially simple, the flexibility and diversification of the cellular responses induced by TGFβ is due to the combinatory interactions with other signals (Guo and Wang, 2009; Luo, 2017). Indeed, SMADs have low and transient DNA-binding affinity, therefore their robust and sustained signaling is exerted through the interaction with several co-transcriptional modulators (Hill, 2016).

Yes-associated protein 1 (YAP, herein referring to YAP1), a transcription co-factor initially discovered as an effector of the Hippo pathway, plays a central role in organ size control via regulation of proliferation and apoptosis in health and disease (Piccolo et al., 2014; Yu et al., 2015; Meng et al., 2016). YAP is crucial in the context of vascular biology. Indeed, it is essential for vascular development, as total YAP knockout mice die ∼8.5 days in to gestation (E8.5) due to defects in yolk sac vasculogenesis (Morin-Kensicki et al., 2006). Moreover, endothelium-specific deletion of YAP leads to impaired atrioventricular cushion formation, a process mainly controlled by EndMT, and embryonic lethality (Zhang et al., 2014). In addition, recent studies demonstrated that YAP controls a wide range of cellular signals, including cell-cell contact (Giampietro et al., 2015), cell polarity (Elbediwy et al., 2016) and mechanical cues (Nakajima et al., 2017), which are all required for the regulation of angiogenesis. Finally, YAP is involved in the morphogenesis, polarization and migration of tip EC and in the proliferation of stalk EC in the developing vasculature (Sakabe et al., 2017; Neto et al., 2018).

YAP is localized both in the cytoplasm and in the nucleus of cells, where it regulates gene transcription (Yagi et al., 1999). Phosphorylation of YAP on Ser127 leads to its sequestration in the cytoplasm (Zhao et al., 2007; Giampietro et al., 2015). As YAP lacks a DNA binding motif, it needs to bind with additional transcriptional factors to signal. Among others, SMAD proteins have been identified (Varelas, 2014; Qin et al., 2018). It has been previously reported that YAP and SMADs signaling converge into an intricate network that governs the activation and maintenance of TGFβ-induced phenotypes (Varelas et al., 2008; Varelas et al., 2010; Dupont et al., 2011).

In the present work, we report that YAP positively contributes to the expression of TGFβ-SMAD3 EndMT early marker genes through two different and combined mechanisms of action. We demonstrate that YAP, by interacting with SMAD3, prevents its binding to GSK3β, thus inhibiting its subsequent phosphorylation and degradation. Moreover, YAP-SMAD3 transcriptional complex triggers the expression of EndMT target genes.

These data support the concept that YAP functions as a co-activator of TGFβ signaling and can be targeted to inhibit EndMT.

## RESULTS

### YAP is a positive regulator of TGFβ-mediated EndMT

**Fig. 1.**
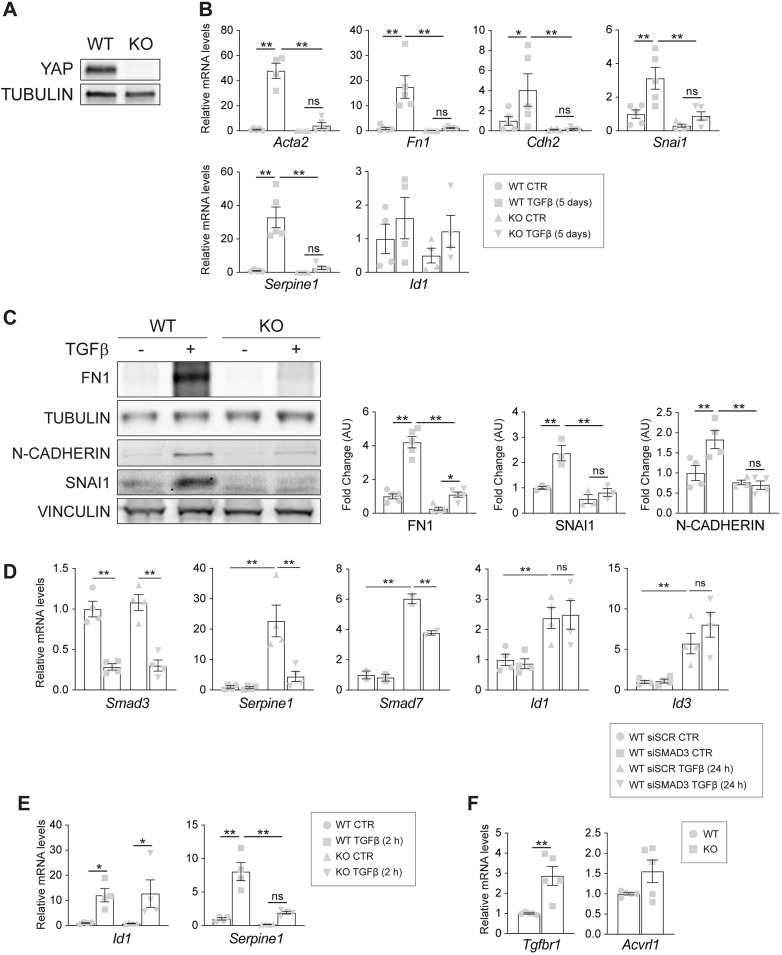
**YAP is a positive regulator of TGFβ-mediated EndMT.** (A) Western blot analysis of YAP expression in wild-type (WT) and knockout (KO) EC. Tubulin was used as a loading control. (B) RT-qPCR analysis of the mesenchymal markers *Acta2*, *Fn1* and *Cdh2*, and of the EndMT-driving transcriptional factors *Snai1*, *Id1* and *Serpine1*. Cells were treated with 5 ng/ml TGFβ for 5 days. Unstimulated cells (CTR) were kept in starving medium for 5 days without the addition of TGFβ. Data are mean±s.e.m. of at least four (*n*≥4) independent experiments. Beta-2 microglobulin (b2m) was used as a housekeeping gene. *P*<0.001 among groups for *Acta2*, *Fn1*, *Snai1*, and *Serpine1*; *P*<0.02 among groups for *Cdh2*; *P*>0.05 for *Id1* (one-way ANOVA). (C) Western blot (left panel) and relative quantification (right panel) of wild-type and knockout cells treated with TGFβ for 5 consecutive days. The blot is representative of *n*=3 independent experiments. Tubulin or Vinculin were used as loading controls. Data are mean±s.e.m. normalized to wild-type untreated cells; *P*<0.001 among groups (one-way ANOVA). (D) RT-qPCR analysis of *Smad3*, *Serpine1*, *Smad7*, *Id1 and Id3* mRNA expression levels in YAP wild-type cells that were transfected with either siSCR or siSMAD3 and treated with 5 ng/ml TGFβ for 24 h. Samples are normalized to wild-type untreated cells. Data are mean±s.e.m. of four (*n*=4) independent experiments. *P*<0.005 among groups (one-way ANOVA). (E) RT-qPCR analysis of *Id1* and *Serpine1* mRNA expression levels in wild-type and knockout cells treated with 5 ng/ml TGFβ for 2 h. Samples are normalized to wild-type untreated cells. Data are mean±s.e.m. of *n*=4 independent experiments. *P*<0.03 among groups for *Id1*; *P*<0.001 among groups for *Serpine1* (one-way ANOVA). (F) RT-qPCR analysis of mRNA expression of *Tgfbr1* (ALK5) and *Acvlr1* (ALK1) in untreated conditions. Data are mean±s.e.m. of five (*n*=5) independent experiments. Beta-2 microglobulin (b2m) was used as a housekeeping gene. **P<0.01 (unpaired *t*-test). Statistical significance was determined using one-way ANOVA (B), Fisher's LSD post-hoc test (B-E) and an unpaired two-tailed Student's *t*-test (F) (*P<0.05, **P<0.01; ns, not significant).

To investigate whether YAP contributes to EndMT in response to TGFβ ligand, we generated and characterized two lung-derived YAP wild-type and knockout EC lines (Fig. 1A). Following immortalization, both cell lines retained their ability to form mature monolayers, as shown by the presence of both adherens and tight junction markers (Fig. S1A), and their endothelial identity (Fig. S1A-C). Moreover, YAP knockout cells showed a lower expression of the tight junction molecule CLAUDIN5 compared to the wild-type counterpart, as previously reported *in vivo* and *in vitro* (Kim et al., 2017). Finally, YAP depletion impaired EC proliferation and migration (Fig. S1D,E) in agreement with previous literature (Zhang et al., 2014; Kim et al., 2017; Neto et al., 2018; Boopathy and Hong, 2019; Hooglugt et al., 2020). These data showed that the established immortalized *in vitro* system, which is a powerful tool for the setup of some experimental conditions and allows a more controlled manipulation of cellular functions and processes, retained the major characteristics of the *in vivo* models and can be used for research, taking into consideration eventual trade-offs (Pan et al., 2009; Lorsch et al., 2014).

We then analyzed the expression of EndMT marker genes upon a chronic treatment with TGFβ for 5 days consecutively (Rudini et al., 2008; Maddaluno et al., 2013). As shown in Fig. 1B**,** TGFβ triggered the expression of EndMT marker genes in wild-type cells, as expected, particularly of the mesenchymal markers *Acta2*, *Fn1*, *Cdh2* and of the early transcription factors involved in EndMT *Serpine1* and *Snai1*. Interestingly, genetic loss of YAP reduced the EndMT response to TGFβ, as knockout cells showed a significant impairment in mRNA upregulation of *Acta2*, *Fn1*, *Cdh2*, *Snai1* and *Serpine1* compared to wild-type treated cells (Fig. 1B). This observation was further verified at the protein level. As shown in Fig. 1C, chronic stimulation by TGFβ strongly increased FN1, N-CADHERIN (encoded by *Cdh2*) and SNAI1 protein expression in wild type but not in knockout cells.

YAP is a key effector of the Hippo pathway together with its paralog protein the transcriptional co-activator with a PDZ-binding domain (TAZ). As an increasing amount of evidence indicated that Hippo coordinates signals triggered by different transduction cascades, such as the TGFβ pathway (Varelas et al., 2008; Hiemer et al., 2014), we investigated whether TAZ was also able to regulate TGFβ-mediated EndMT in a similar way. Interestingly, lentiviral knockdown of TAZ expression (Fig. S2A), which was sufficient to significantly inhibit the expression of YAP/TAZ direct target genes (Basu-Roy et al., 2015; Choi et al., 2015; Venkataramani et al., 2018) (Fig. S2B), did not affect EndMT marker gene expression, neither in wild-type nor in YAP knockout cells, and did not further decrease EndMT marker levels induced by chronic treatment with TGFβ (Fig. S2C), suggesting that, in this context, the two paralogs have no compensatory, redundant or additive effects. Of note, in this cell system, YAP is expressed at a higher level compared to TAZ (Fig. S2), so it cannot be excluded that this difference of expression has an effect on the regulation of the TGFβ pathway.

Finally, to rule out the possibility that YAP was directing TEAD-mediated transcription of TGFβ-responsive genes as previously reported in other model system (Marquard et al., 2020), we silenced TEAD1 and analyzed the expression levels of some EndMT markers upon TGFβ-stimulation (24 h). As shown in Fig. S3, TEAD1 downregulation, which was sufficient to significantly inhibit the expression of its direct target genes, did not significantly affect EndMT marker gene expression in any tested condition. All these data show that YAP, and not TAZ, is a specific positive regulator of TGFβ-mediated EndMT in a TEAD-independent manner.

### YAP is specifically required for SMAD3 signaling in EC

TGFβ primarily signals through receptor-associated SMADs (R-SMADs) (Hu et al., 2018), among which SMAD3 is considered the canonical effector of TGFβ (Goumans and Ten Dijke, 2017). We checked the expression levels of *Serpine1* and *Smad7* canonical targets of ALK5/SMAD3 signaling, and *Id1* and *Id3* canonical target of ALK1/SMAD1 signaling, (Dennler et al., 1998; Goumans et al., 2002), respectively, upon *Smad3* downregulation in wild-type cells. Chronic TGFβ treatment upregulated the level of all these genes, whereas *Smad3* depletion specifically impaired the upregulation of *Serpine1* and *Smad7* (Fig. 1D).

We then evaluated SMAD transcriptional activity at earlier time points. In line with the chronic stimulation, acute TGFβ treatment induced *Id1* upregulation at a similar level in both cell lines, and *Serpine1* expression was significantly affected in the absence of YAP (Fig. 1E). To corroborate these results, we performed the experiment using primary cells. As reported in Fig. S4, in line with what was obtained with immortalized cell lines, upon TGFβ stimulation, *Serpine1* induction was reduced in YAP-depleted samples. These results suggest that TGFβ downstream signaling is impaired in the absence of YAP and that YAP is specifically required for SMAD3 signaling in EC.

### YAP is not required for the initial steps of TGFβ signaling activation

TGFβ ligands signal through a heteromeric complex of receptors (Hata and Chen, 2016). We thus verified the expression levels of ALK5 (also known as Tgfbr1), the receptor responsible for SMAD2 and SMAD3 phosphorylation and activation in EC, and of ALK1 (also known as Acvlr1), responsible for SMAD1, SMAD5 and SMAD8 phosphorylation and activation in EC (Goumans and Ten Dijke, 2017). Quantitative analysis revealed a slight increase rather than a decrease in the expression of both receptors in YAP knockout cells (Fig. 1F), suggesting that the expression of EndMT markers is not impaired by a reduction of the expression of the two main type I TGFβ receptors (TβRI).

**Fig. 2.**
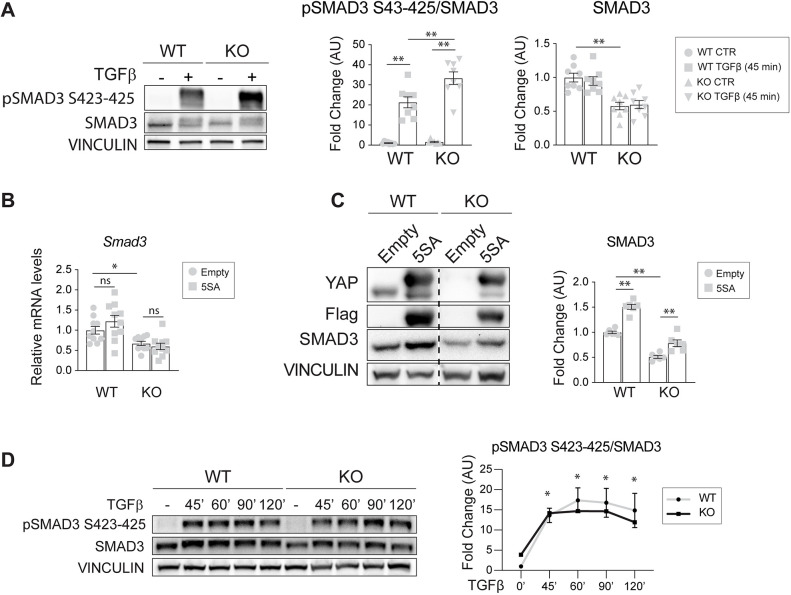
**YAP contributes to SMAD3 signaling in EC.** (A) Representative western blot of C-term phosphorylated SMAD3 (pSMAD3 S423-425) protein in wild-type (WT) and knockout (KO) cells treated with TGFβ for 45 min, and relative total SMAD3 expression levels. Vinculin bands represent the loading control. The ratio between normalized pSMAD3 S423-425/SMAD3 levels is expressed as fold change referring to wild-type untreated cells in arbitrary units (AU). *n*=8 (pSMAD3/SMAD3 independent experiment). *P*<0.001 among groups for pSMAD3/SMAD3, SMAD3 (one-way ANOVA). (B) RT-qPCR analysis of *Smad3* mRNA expression levels in YAP wild-type and knockout cells that were infected with either empty or YAP 5SA lentiviral vectors. Samples are normalized to wild-type empty cells. *n*=10 independent experiments. *P*<0.001 among groups (one-way ANOVA). (C) Representative western blot (left) of YAP wild-type and knockout cells that were infected with either empty or YAP 5SA lentiviral vectors, and quantification (right). Vinculin represents the loading control. The dashed line separates two different exposures of the same membrane. The fold change refers to wild-type empty cells. *n*=6 independent experiments. *P*<0.001 among groups (one-way ANOVA), (D) Representative western blot and quantification of YAP wild-type and knockout cells treated with TGFβ for the indicated times. Vinculin represents the loading control. The ratio between normalized pSMAD3 S423-425 and SMAD3 levels is expressed as fold change referring to wild-type untreated cells in arbitrary units. *n*=3 independent experiments. Data are mean±s.e.m. pSMAD3/SMAD3: *P*<0.001 among time points, *P*>0.05 between genotypes (two-way ANOVA); *P<0.001 both wild-type and knockout versus their counterpart at 0 min (0′). Statistical significance was determined using one-way ANOVA (A-D) and Fisher's LSD post-hoc test (B-D) (*P<0.05, **P<0.01; ns, not significant).

Upon TGFβ stimulation, TβRI phosphorylate R-SMADs at their C terminus (C-term, S423-4225) (Goumans and Ten Dijke, 2017). We then tested SMAD3 C-term phosphorylation upon 45 min of TGFβ stimulation. The treatment induced a strong and significant C-term phosphorylation of SMAD3 in both wild-type and knockout cells (Fig. 2A). Interestingly, this increase was higher in knockout cells, similar to the level of the two TβRI receptors (Fig. 1F). We concluded that the lack of YAP expression does not affect the initial activation of the TGFβ signaling pathway. Interestingly, in the absence of YAP there was a significant reduction of total SMAD3 protein levels (Fig. 2A), suggesting that YAP controls SMAD3 expression and subsequent signaling activity.

Therefore, we introduced in our model system a constitutively transcriptionally active form of flagged-YAP (YAP 5SA), which cannot be phosphorylated in any of the five serine residues required for YAP cytoplasmic retention and therefore localizes predominantly in the nucleus (Zhao et al., 2007; Dupont et al., 2011). The expression of YAP 5SA in knockout cells did not rescue SMAD3 mRNA expression (Fig. 2B) but increased SMAD3 protein expression in both cell lines (Fig. 2C), indicating that YAP is not directly involved in *Smad3* gene transcription but is perhaps involved in SMAD3 protein stabilization.

Finally, to test whether the absence of YAP might result in a faster SMAD3 C-term dephosphorylation and, thus, in a quicker SMAD3 signaling shutdown, we performed a TGFβ time course and checked the C-term phosphorylation levels of SMAD3 over time. Compared to the untreated controls, SMAD3 phosphorylation occurred with comparable kinetics in the two cell lines, suggesting that YAP did not influence SMAD3 C-term phosphorylation (Fig. 2D). Collectively, these results show that YAP is not required for the initial steps of TGFβ signaling activation.

### YAP controls SMAD3 localization into the nucleus

As our data showed that YAP is necessary for SMAD3-driven signaling activity (Fig. 1), but not for TGFβ-induced cascade activation (Fig. 2), we investigated whether YAP is involved in the nuclear accumulation of downstream R-SMADs. Following receptor-mediated phosphorylation, R-SMADs form a complex with SMAD4 and together shuttle to the nucleus (Goumans and Ten Dijke, 2017).

**Fig. 3.**
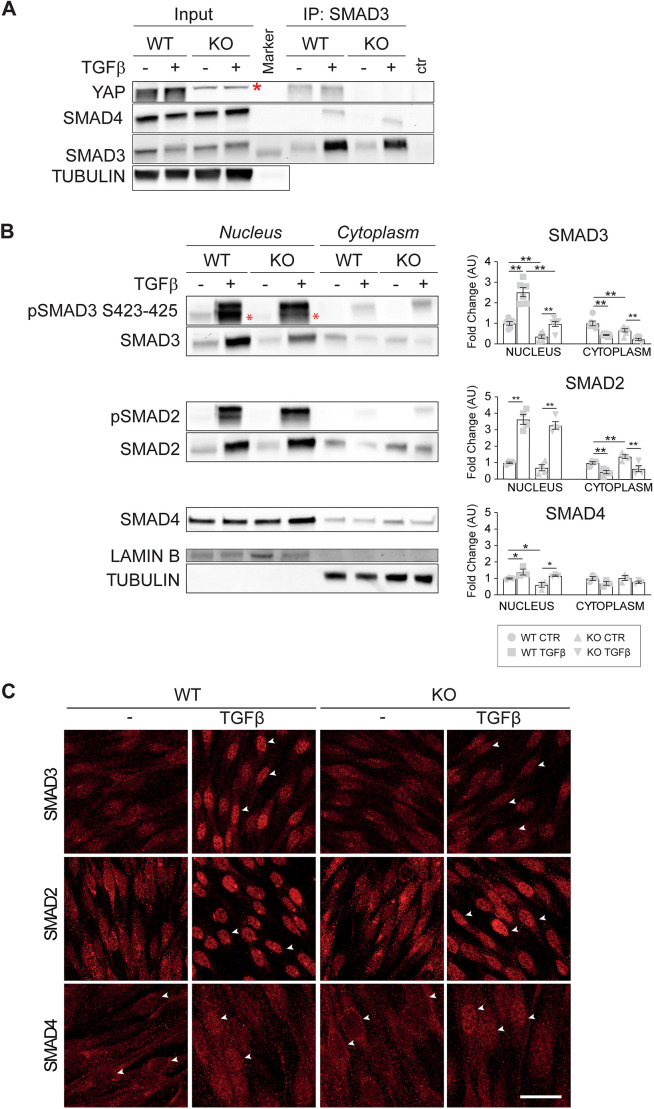
**YAP controls SMAD3 localization into the nucleus.** (A) Representative immunoprecipitation (IP) of SMAD3 and western blot for SMAD3, SMAD4 and YAP on YAP wild-type (WT) and knockout (KO) cells treated with TGFβ for 45 min. The asterisk indicates an unspecific band detected in knockout cells. Tubulin was used as the loading control. (B) Representative western blot of nuclear-cytoplasmic fractionation after 2 h of TGFβ treatment and quantification. Red asterisks in the blot indicate the specific pSMAD3 S423-425 band. Data are mean±s.e.m. of at least three (*n*≥3) independent experiments. *P*<0.001 among groups except for SMAD4 cytoplasm (*P*>0.05; one-way ANOVA). *P*<0.05, **P<0.01 (Fisher's LSD post-hoc test). Fold changes refer to either nuclear or cytoplasmic wild-type untreated cells. Lamin B was used as the loading control for the nuclear fraction. Tubulin was used as loading control for the cytoplasmic fraction and to verify the purity of the nuclear fraction. (C) Representative immunofluorescence staining of total SMAD3, SMAD2 and SMAD4 in wild-type and knockout cells either treated or not with TGFβ for 2 h. White arrowheads point to nuclei. Scale bar: 20 µm. AU, arbitrary units; CTR, control.

Immunoprecipitation analyses from total cell extracts of wild-type and knockout EC showed that SMAD3 binds SMAD4 in both cell lines upon TGFβ stimulation, indicating that loss of YAP does not affect SMAD3-SMAD4 complex formation (Fig. 3A). Moreover, these analyses revealed that SMAD3 formed a protein complex together with YAP in both control and stimulated conditions.

We then treated wild-type and knockout cells for 2 h with TGFβ, and analyzed SMAD subcellular localization to determine whether YAP is relevant for R-SMADs nuclear accumulation. A nuclear-cytoplasmic fractionation assay (N/C) (Fig. 3B) and immunofluorescence analyses (Fig. 3C) showed that endothelial stimulation with TGFβ induced a marked SMAD3 and SMAD2 (but not SMAD4) nuclear relocalization in wild-type cells, and loss of YAP significantly and specifically affected SMAD3 nuclear accumulation. These results, in agreement with previous studies (Zhang et al., 2014), showed that YAP is required for both SMAD3 protein accumulation (Fig. 2A) and nuclear translocation (Fig. 3), and, overall, for SMAD3 signaling activity (Fig. 1), further confirming that YAP and SMAD3 cooperate to drive TGFβ-induced signaling in EC.

**Fig. 4.**
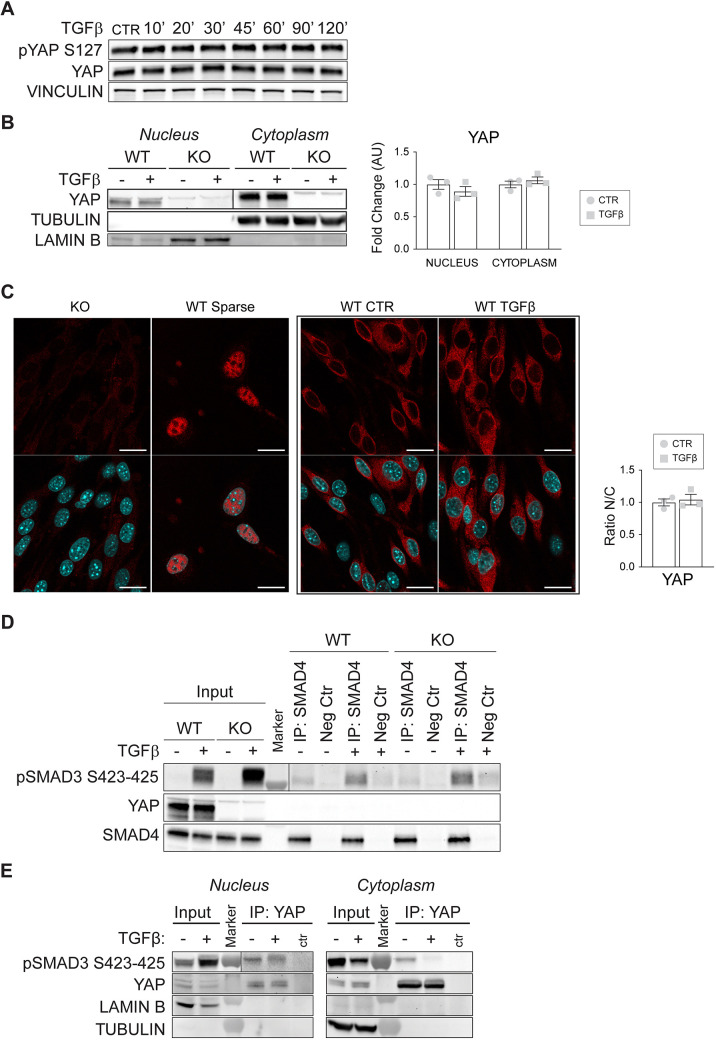
**YAP subcellular localization is not modulated by TGFβ, and YAP does not contribute to SMAD4-mediated SMAD3 nuclear translocation.** (A) Representative western blot of pYAP S127 level in YAP wild-type (WT) cells treated with TGFβ for the indicated times. Vinculin was used as the loading control. (B) Representative western blot of nuclear-cytoplasmic fractionation after 2 h of TGFβ treatment (left), and quantification (right). Fold changes refer to either nuclear or cytoplasmic YAP wild-type untreated cells. Lamin B was used as the loading control for the nuclear fraction. Tubulin was used as a loading control for the cytoplasmic fraction and to verify the purity of the nuclear fraction. (C) Immunostaining of YAP in wild-type confluent cells after 2 h of TGFβ treatment (left), and quantification (right). YAP knockout (KO) cells were used as a negative control for the staining; sparse wild-type cells were used as positive control of nuclear localization of YAP. Scale bars: 20 µm. For the quantification of the ratio between the nuclear and the cytoplasmic intensity of the staining in wild-type cells treated or not with TGFβ, samples are normalized to YAP wild-type untreated cells. (D) Representative (*n*=3) immunoprecipitation (IP) of SMAD4 and western blot for SMAD4, YAP and pSMAD3 S423-425 of YAP wild-type and knockout cells treated with TGFβ for 2 h before performing immunoprecipitation (see Materials and Methods). As a negative control (Neg Ctr), a species-matching antibody was used. (E) Representative western blot of wild-type cells immunoprecipitated for YAP from either the nuclear or cytosolic cell fraction. Cells were treated with TGFβ for 45 min, *n*=3 independent experiments. Vertical lines in B, D and E separate two different exposures of the same membrane. Data are mean±s.e.m. of three independent experiments. *P*>0.05 (unpaired two-tailed Student's *t*-test). AU, arbitrary units; CTR, control.

In conclusion, loss of YAP did not impair SMAD3 and SMAD4 complex formation, but it strongly reduced the amount of SMAD3 that accumulates in the nucleus upon TGFβ treatment. In order to define how YAP contributes to this process, we first checked whether YAP subcellular localization varied upon TGFβ treatment. We performed TGFβ time-course treatment of wild-type cells and analyzed over time the phosphorylation of YAP on Ser127, which is responsible for its cytoplasmic retention in both canonical and non-canonical Hippo signaling pathways (Basu et al., 2003; Zhao et al., 2007; Giampietro et al., 2015; Moon et al., 2017). Interestingly, we found that TGFβ did not affect YAP phosphorylation at any time point tested (Fig. 4A), suggesting that YAP does not shuttle to the nucleus in response to TGFβ stimulation.

To strengthen this evidence, we then performed both N/C and immunofluorescence analyses to check YAP subcellular localization in the presence of a TGFβ stimulus (Fig. 4B-D). In line with our previous observations, and with other cell system studies (Varelas et al., 2010; Giampietro et al., 2015), in confluent endothelial monolayers YAP mostly localizes in the cytoplasm (confluent YAP knockout and sparse YAP wild-type cells were negative and positive control for the staining and nuclear localization, respectively), although a small amount of YAP was present in the nucleus. Moreover, no YAP nuclear accumulation was detected upon TGFβ treatments, thus indicating that TGFβ did not modulate YAP subcellular localization.

Finally, by performing immunoprecipitation of SMAD4 from total cell lysate in the presence or absence of a TGFβ stimulus, no interaction between SMAD4 and YAP was detected (Fig. 4E). These data, together with the previous observation that SMAD3 binds YAP (Fig. 3A), suggest that SMAD3, SMAD4 and YAP do not form a trimeric complex, and that SMAD4 and YAP binding to SMAD3 is mutually exclusive. Collectively, all of these data show that YAP subcellular localization is not modulated by TGFβ and that YAP does not contribute to SMAD4-mediated SMAD3 nuclear translocation. Immunoprecipitation of YAP from N/C upon TGFβ stimulation was performed. Interestingly, YAP and SMAD3 interacted with each other both in the nucleus and in the cytoplasm (Fig. 4E).

In light of these results, we hypothesize that YAP, by acting as a transcriptional co-regulator, interacts with SMAD3 in the nucleus, strengthening SMAD3 binding to the DNA and positively regulating EndMT gene expression. At the same time, this binding could prevent SMAD3 nuclear exit and degradation, thus in turn increasing SMAD3 protein level.

### YAP prevents SMAD3 phosphorylation and turnover

The data presented here suggest that YAP plays this double role sustaining SMAD3 transcriptional activity while preventing its turnover. As reintroducing a transcriptionally constitutively active form of YAP resulted in a marked increase of SMAD3 protein in knockout cells (Fig. 2C), we hypothesized that YAP functioned as SMAD3 co-regulator to drive EndMT gene transcription (Fig. 1B) while preventing its phosphorylation at S204, which is responsible for its subsequent degradation (Gao et al., 2009; Wang et al., 2009).

**Fig. 5.**
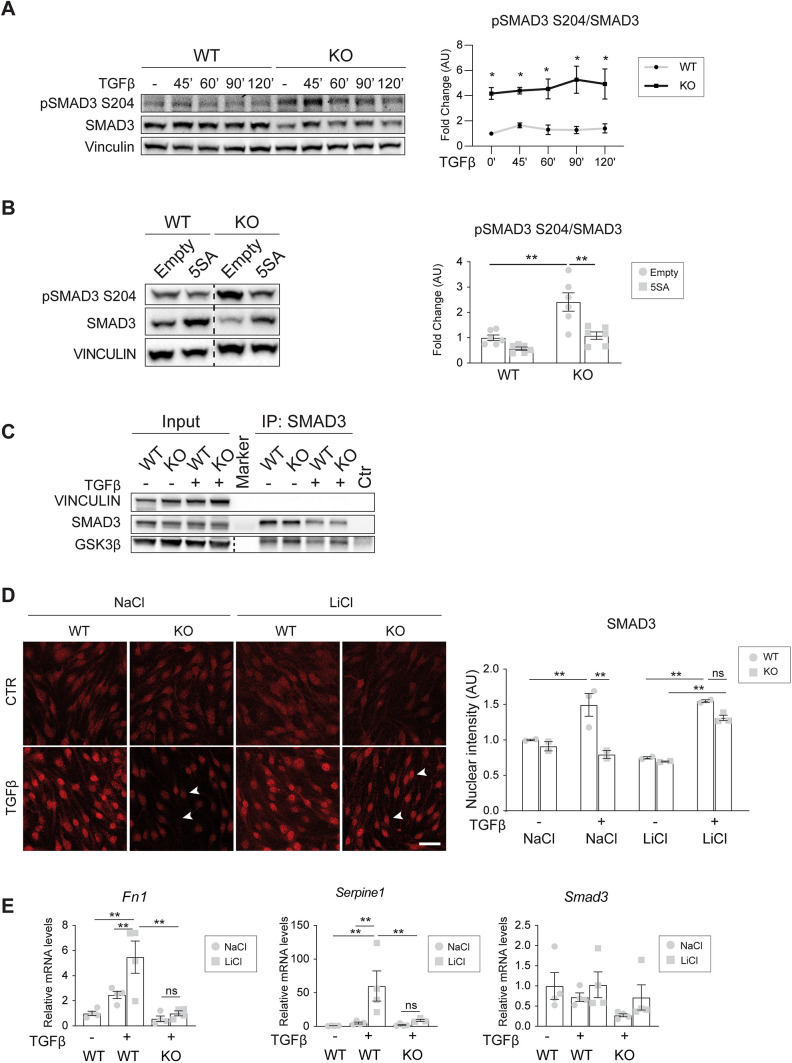
**YAP prevents SMAD3 phosphorylation, turnover and SMAD3-GSK3β binding.** (A) Representative western blot and quantification of YAP wild-type (WT) and knockout (KO) cells treated with TGFβ for the indicated times. Vinculin was used as the loading control. The ratio between normalized pSMAD3 S204 and SMAD3 levels were expressed as fold change referring to wild-type untreated cells in arbitrary units (AU). *n*=3. *P*>0.05 among time points. *P*<0.005 between genotypes (two-way ANOVA); *P<0.01 versus wild type (Fisher's LSD post-hoc test). (B) Representative western blot and quantification of YAP wild-type and knockout cells infected with either empty or YAP 5SA lentiviral vectors. Vinculin was used as the loading control. The fold change refers to wild-type empty cells. *n*=6. *P*<0.001 among groups (one-way ANOVA); **P<0.001 (Fisher's LSD post hoc test). (C) Immunopreciptation (IP) SMAD3 and western blot for SMAD3 and GSK3β on YAP wild-type and knockout cells treated with TGFβ for 45 min before performing immunoprecipitation. Vinculin was used as the loading control of input. (D) Representative immunofluorescence (IF) of total SMAD3 in wild-type and knockout cells treated with either 60 mM LiCl or NaCl (control) overnight in starving medium, followed by 2 h TGFβ stimulation. Arrowheads indicate nuclei. Scale bar: 40 µm. The plot on the right represents the mean intensity of nuclear SMAD3±s.e.m. from two or three independent experiments, expressed as a fold change referring to wild-type NaCl-untreated cells. *P*<0.001 among groups (one-way ANOVA). *P*<0.001 among groups; **P<0.01 (Fisher's LSD post-hoc test). (E) RT-qPCR of *Fn1*, *Serpine1* and *Smad3* mRNA expression levels in YAP wild-type and knockout cells treated with either 60 mM LiCl or NaCl (control) overnight in starving medium, followed by 24 h TGFβ stimulation. Samples are normalized to wild-type NaCl-untreated cells. *N*=4. Data are mean±s.e.m. *P*<0.01 among groups except for *Smad3* (*P*>0.05). Statistical significance was determined using one-way ANOVA and Fisher's LSD post-hoc test (*P<0.05, **P<0.01; ns, not significant). Dashed lines in B and C separate two different exposures of the same membrane. Ctr, control.

Thus, we verified whether lack of YAP expression resulted in an increased phosphorylation of SMAD3 at S204 (Fig. 5A). Interestingly, TGFβ time-course treatment revealed that SMAD3 was phosphorylated at a higher level in YAP knockout versus wild-type cells already at basal conditions, suggesting that YAP expression prevented SMAD3 pS204 even in the absence of a TGFβ stimulus. Consistently, YAP 5SA expression in knockout cells resulted in a marked decrease of pSMAD3 S204 (Fig. 5B). Together these results indicate that YAP, likely by engaging SMAD3 as a co-regulator, limits its phosphorylation at S204 and its turnover.

### YAP prevents SMAD3-GSK3β binding

We then investigated whether GSK3β is the kinase responsible for the phosphorylation of SMAD3 at S204. Immunoprecipitation showed that SMAD3 and GSK3β form a complex in EC (Fig. 5C). We then analyzed SMAD3 nuclear accumulation after inhibiting GSK3β activity, and thus protein degradation, with LiCl treatment (Klein and Melton, 1996). In response to TGFβ, we observed a significant increase in SMAD3 nuclear accumulation in knockout cells treated with LiCl compared to knockout cells treated with NaCl as a negative control (Fig. 5D), suggesting that inhibition of GSK3β-mediated SMAD3 protein turnover is sufficient to restore SMAD3 nuclear accumulation in knockout cells.

Given these results, we reasoned whether the defective EndMT response of YAP knockout cells (Fig. 1) was only due to decreased levels in SMAD3 protein expression and nuclear accumulation, or whether, for the EndMT response to take place, SMAD3 required YAP as an active transcriptional co-regulator. To address this question, we analyzed EndMT response in wild-type and knockout cells stimulated with TGFβ and treated with LiCl. Interestingly, inhibition of GSK3β significantly upregulated *Fn1* and *Serpine1* mRNA expression in wild type but not in knockout cells in response to TGFβ (Fig. 5E). Moreover, LiCl treatments did not have any effect on SMAD3 mRNA expression levels, further confirming that GSK3β inhibition played a role in SMAD3 protein stabilization.

### YAP is a transcriptional co-factor that drives SMAD3-mediated EndMT program

These results strongly suggested that YAP was also required as a SMAD3 transcriptional co-regulator to trigger the EndMT program. To determine whether YAP and SMAD3 cooperate as co-factors in driving EndMT gene expression, we analyzed the promoter region of several EndMT genes, spanning −5.0 kb to +1.0 kb around the transcription start site.

Analyses revealed that the promoter regions of the mesenchymal marker *Fn1*, along with the early EndMT-driving transcription factor *Snai1* and *Serpine1*, contain SMAD3 putative binding sites, (Fig. S5A). It was previously reported that YAP and SMAD3 mediate *Snai1* transcription by binding to its promoter region in response to 2 h of TGFβ treatment (Zhang et al., 2014). Thus, we focused our attention on the *Fn1* gene, as its expression is highly upregulated in the presence of YAP upon TGFβ stimulation (Fig. 1C) and it has a well-established role in epithelial-to-mesenchymal transition (EMT) (Câmara and Jarai, 2010; Li et al., 2017; Bulzico et al., 2019; Li et al., 2019).

**Fig. 6.**
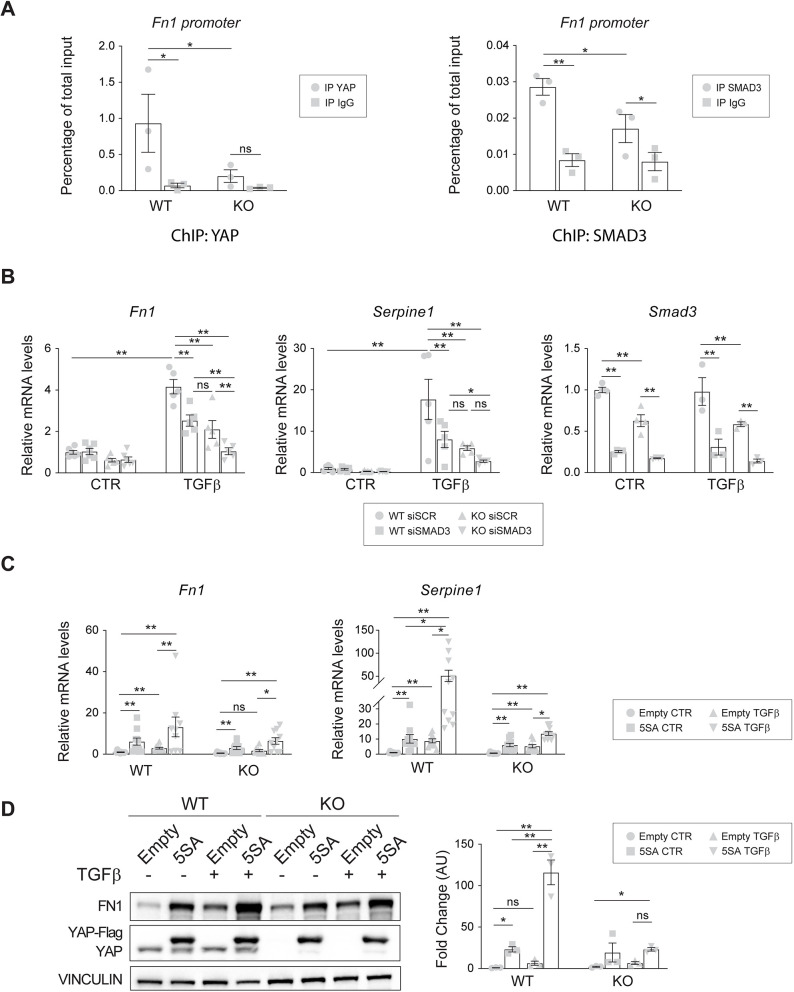
**YAP is a SMAD3 transcriptional co-factor for driving EndMT program.** (A) ChIP qPCR analysis of YAP (left) and SMAD3 (right) binding to the *Fn1* promoter. Wild-type (WT) and knockout (KO) cells were treated for 6 h with TGFβ. DNA levels are normalized to the relative inputs. *n*=3 independent experiments. *P*<0.05 among groups for YAP and *P*<0.003 for SMAD3 (one-way ANOVA). (B) RT-qPCR analysis of *Fn1*, *Serpine1* and *Smad3* mRNA expression levels in YAP wild-type and knockout cells that were transfected with either siSCR or siSMAD3 and treated with 5 ng/ml TGFβ for 24 h. Samples are normalized to wild-type untreated cells. *n*=4-6 independent experiments. *P*<0.0001 among groups (one-way ANOVA). (C) RT-qPCR analysis of *Fn1* and *Serpine1* mRNA expression levels in YAP wild-type and knockout cells that were infected with either empty or YAP 5SA lentiviral vectors and treated with 5 ng/ml TGFβ for 24 h. Samples are normalized to wild-type empty untreated cells. *n*=10 independent experiments. (D) Representative western blot and quantification of YAP wild-type and knockout cells that were infected with either empty or YAP 5SA lentiviral vectors and treated with 5 ng/ml TGFβ for 24 h. *n*=3 independent experiments. *P*<0.0001 among groups (one-way ANOVA). Vinculin was used as the loading control. The fold change refers to wild-type empty untreated cells. Data are mean±s.e.m. Statistical significance was determined using one-way ANOVA (A,B,D), Fisher's LSD post-hoc test, Kruskal–Wallis test (C, *P*<0.001 among groups) and Dunn's post-hoc test (C). (*P<0.05; **P<0.01; ns, not significant). AU, arbitrary units; CTR, control.

Having established that YAP and SMAD3 form a complex (Fig. 4E), we next assessed whether YAP and SMAD3 act as transcriptional regulators of TGFβ-induced *Fn1* expression by binding the identified putative binding site (Fig. S5A). We performed ChIP qPCR assays of wild-type and knockout cells stimulated with TGFβ for 6 h, as that is the earliest time point at which *Fn1* expression is significantly upregulated (Fig. S5B). Interestingly, both YAP and SMAD3 bound to the *Fn1* promoter region at the level of a SMAD3 putative binding site, suggesting that they possibly worked as *Fn1* transcriptional regulators (Fig. 6A). Interestingly, in the absence of YAP, the binding of SMAD3 on the promoter was significantly reduced, suggesting that YAP stabilizes/strengthens the binding of SMAD3. To further support these results, we silenced SMAD3 in both wild-type and knockout cells and analyzed the expression of *Fn1*, as well as *Serpine1* as an internal control (Fig. 6B), in response to TGFβ treatment. The results showed that SMAD3 silencing affected the TGFβ induction of *Fn1* in both cell lines. These data showed that both YAP and SMAD3 induce *Fn1* expression upon TGFβ stimulation, suggesting that they co-operate to drive EndMT.

To strengthen this evidence, YAP 5SA infected cells (Fig. 2C) were stimulated with TGFβ for 24 h and the EndMT response was assessed in terms of *Fn1* and *Serpine1* mRNA expression. Consistent with previous results, YAP 5SA strongly induced *Fn1* and *Serpine1* expression in both wild-type and knockout cells already at basal levels, suggesting that active nuclear YAP is important for the transcription of these genes (Fig. 6C). Moreover, TGFβ treatment further increased *Fn1* and *Serpine1* expression in wild-type and knockout YAP gain-of-function cells (YAP 5SA), leading to comparable EndMT gene transcription levels between KO 5SA- and wild-type empty control cells. Of note, YAP 5SA expression in knockout cells did not upregulate the expression of EndMT genes at the same level as in wild-type 5SA TGFβ-treated cells, suggesting that nuclear YAP activity is not enough to fully rescue SMAD3-driven signaling. This observation was further verified at the protein level (Fig. 6D). Collectively, these results show that both YAP and SMAD3 bind to the *Fn1* promoter region and that YAP can function as SMAD3 co-regulator to drive EndMT.

## DISCUSSION

EC exposed to different milieus undergo dynamic phenotypic switching, a key process in the context of endothelial heterogeneity that, when deregulated, might result in EC dysfunction. Evidence shows the importance of EndMT in endothelial dysfunction of cardiovascular diseases and cancer (Potenta et al., 2008; Ha et al., 2018; Kovacic et al., 2019). EndMT is a complex biological process in which EC progressively lose some endothelial characteristics and acquire mesenchymal phenotype. The TGFβ signaling pathway is the most well-known EndMT inducer but in the context of vascular biology emerging evidence demonstrates a crucial role also for YAP (Zhang et al., 2014).

Several works have reported a crosstalk between YAP and the TGFβ signaling pathway in physiological, as well as pathological, conditions (Hiemer et al., 2014; Zhang et al., 2014; Piersma et al., 2015). YAP binds TGFβ-activated SMAD complexes to control SMAD cellular localization and activity in many cell types, including epithelial cells (Varelas et al., 2010; Pefani et al., 2016; Liu et al., 2017) and fibroblasts (Szeto et al., 2016; Qin et al., 2018). Moreover, it has been shown that the transcriptional activity of the YAP-TEAD-SMADs complex controls the maintenance of human embryonic stem cell pluripotency (Beyer et al., 2013). Thus, YAP can modify TGFβ pathway activation through the interaction with different SMADs.

Previous studies have reported YAP as an important promoter of EMT (Overholtzer et al., 2006; Yuan et al., 2016). Our group and others have shown that chronic exposure to the TGFβ/BMP family of ligands strongly correlates with physiological and pathological EndMT (Azhar et al., 2009; Medici et al., 2010; Maddaluno et al., 2013; Malinverno et al., 2019). Finally, it has been shown that the endothelial-specific deletion of YAP prevents the proper endocardial cushion formation in mouse embryo (Zhang et al., 2014), a process mainly driven by EndMT (Camenisch et al., 2002; Sugi et al., 2004; Ma et al., 2005; Yamagishi et al., 2009).

The data presented here reveal a dual mechanism of cross-talk between YAP and the TGFβ signaling pathway that regulates early EndMT, which specifically involves SMAD3. By comparing lung-derived EC from adult mice wild type and YAP knockout lines, we found that upon TGFβ treatment in the absence of YAP the expression of EndMT markers, such as *Acta2*, *Fn1*, *Cdh2*, *Snai1* and *Serpine1*, was strongly limited. These data are consistent with the observation that *in vivo* endothelial-specific YAP knockout mice resulted in defective EndMT during the atrioventricular cushion formation (Zhang et al., 2014).

YAP and TAZ proteins are transcriptional co-regulators encoded by paralogous genes, which are activated in response to multiple inputs (Pocaterra et al., 2020). In the cardiovascular system, the concurrent inhibition of YAP and TAZ caused defects in the formation of coronary vasculature due to abnormal cell proliferation and deregulated EMT in epicardial cells (Singh et al., 2016). Interestingly, Neto et al. (2018) demonstrated that endothelial-specific deletion of YAP or TAZ led to mild vascular defects, suggesting compensatory regulation, whereas the deletion of both proteins led to a dramatic defect in retinal blood vessel development.

Here, we showed that EndMT impairment was due to a specific loss of YAP, whereas its paralog TAZ did not have any role in this context. These results are in line with previous studies showing that there are differences in the role of YAP and TAZ (Morin-Kensicki et al., 2006; Hossain et al., 2007; Tian et al., 2007; Makita et al., 2008), and that they are not completely redundant as, under certain conditions, TAZ is unable to compensate for the loss of YAP (Neto et al., 2018; Plouffe et al., 2018). Functions of YAP have been mainly attributed to its interaction with the TEAD family of transcription factors (Zhao et al., 2008; Zhang et al., 2009; Liu-Chittenden et al., 2012; Walko et al., 2017). As TEAD1 is a primary mediator of YAP1-dependent gene regulation (Ota and Sasaki, 2008; Stein et al., 2015), and YAP-TEAD1 signaling has been shown to control angiogenesis (Mammoto et al., 2018), we looked for a role for TEAD1 and found that it was not involved in this mechanism, although a more general TEAD-dependent effect cannot be excluded.

These findings raise the question about the molecular mechanisms driving the crosstalk between YAP and the TGFβ signaling pathway. YAP did not affect the initial steps of TGFβ signaling activation. Indeed, the expression levels of the TβRI receptors, as well as the C-term phosphorylation levels of SMAD3, were not reduced in YAP knockout cells. Conversely, SMAD3 nuclear localization was reduced in the absence of YAP, as well as SMAD3 mRNA and protein levels, opening up the possibility that YAP could regulate SMAD3 transcription and, in this way, TGFβ-mediated EndMT. However, by a gain-of-function approach, reintroducing a transcriptionally active form of YAP (YAP 5SA) in knockout cells, we did not observe any rescue in SMAD3 mRNA transcription, suggesting that SMAD3 transcription is not directly regulated by YAP, rather YAP 5SA expression led to a significant upregulation of SMAD3 protein levels.

We also tested the hypothesis that nuclear YAP prevents SMAD3 protein turnover. Previous studies analyzed this phenomenon: upon C-term phosphorylation, SMAD3 relocalizes into the nucleus where it is further phosphorylated by CDK8/9 in its linker region first at T179. Phosphorylation of the T179 residue allows the binding of co-regulators and target gene transcription (Alarcón et al., 2009). In epithelial cells, the binding of transcription co-factors to pT179-SMAD3 prevents SMAD3 subsequent phosphorylation on S204 by GSK3β and proteasomal degradation (Gao et al., 2009; Wang et al., 2009). In this way, although ensuring target gene transcription, binding of transcription co-factors to SMAD3 inhibits its degradation (Alarcón et al., 2009; Aragon et al., 2011). In our model, the levels of SMAD3 pS204, the phosphorylation described as targeting SMAD3 for protein degradation (Aragon et al., 2011), were strongly increased in the absence of YAP. Moreover, YAP 5SA expression markedly reduced SMAD3 pS204 levels in knockout cells, indicating that nuclear YAP prevents SMAD3 S204 phosphorylation upon TGFβ treatment. A previous report showed that SMAD3 S204 can be phosphorylated by GSK3β in response to TGFβ (Wang et al., 2009). Consistently, we found higher amounts of GSK3β bound to SMAD3 in the absence of YAP. Finally, the inhibition of GSK3β activity by LiCl treatment was capable of increasing total SMAD3 protein level accumulation, but this was insufficient to allow transcription of the EndMT genes in TGFβ-stimulated YAP knockout cells. All of these findings are in line with the hypothesis that nuclear YAP could play a double role in SMAD3 signaling activity, functioning at the same time as SMAD3 co-regulator while preventing its turnover in response to TGFβ. Indeed, we found that both SMAD3 and YAP bound to the same region on the *Fn1* promoter and that the absence of YAP reduced the binding of SMAD3, suggesting that they work together to drive the expression of this gene.

Overall, these data extend the knowledge on the complex mechanism of crosstalk between YAP and the TGFβ pathway. We proved that YAP is an important positive regulator of TGFβ-mediated EndMT by specifically sustaining SMAD3-driven signaling activity. The identified mechanism opens novel therapeutic opportunities to treat pathological conditions in which EndMT occurs, such as tumor metastatization or organ fibrosis (Zeisberg et al., 2007a,b, 2008; Fujii et al., 2012; Hiemer et al., 2014).

Besides the above mentioned pathophysiological relevance, these findings are important because of their possible connection to other receptor-mediated pathways. Indeed, it has been shown that YAP is a central mediator of VEGF signaling in endothelial cells in which it contributes to angiogenesis (Wang et al., 2017; Azad et al., 2018; Elaimy and Mercurio, 2018), as well as in tumor cells (Zanconato et al., 2016; Elaimy and Mercurio, 2018). As both VEGF and TGFβ signaling pathways are tightly regulated during development and aberrantly activated in vascular pathologies, the impact of this work is likely to add to the interest in targeting YAP as a therapeutic approach to inhibit converging molecular pathways.

## MATERIALS AND METHODS

### Cell isolation, immortalization and culture

Lung EC were isolated and immortalized, as described previously (Dong et al., 1997), from a YAPf/f mouse (Donato et al., 2018). Briefly, mouse lungs were removed under sterile conditions, washed two times with PBS and minced finely with scalpels. Organ disaggregation was carried out by incubating minced lungs with collagenase A (1.5 mg/ml; Roche) and DNAse (25 μg/ml; Roche) in Dulbecco's modified Eagle medium (DMEM; 37°C for 3 h). After filtering through a nylon screen, cells were collected, centrifuged at 300 ***g*** for 10 min and then seeded in 0.1% gelatin-coated 24-well plates. Then, 48 h afterwards, EC were washed with PBS and infected with polyoma middle T antigen supernatant to specifically select and immortalize only EC. The supernatant was then replaced with complete medium after 8 h. After 3 months in culture, we obtained a homogeneous population of EC, the purity of which was analyzed by performing extensive staining for endothelial-specific molecules. Yap floxed alleles were deleted by treating pure EC *in vitro* with TAT-Cre recombinase using Hyclone ADCF-Mab medium (Thermo Scientific), as described previously (Liebner et al., 2008), thus generating YAP wild-type and knockout immortalized lung EC lines. Lung EC were grown on 1% gelatin-coated plates in complete medium, containing MCDB-131 (GIBCO) supplemented with 20% South American fetal bovine serum (FBS) (HyClone), penicillin/streptomycin (100 units/L; Sigma-Aldrich), sodium pyruvate (1 mM), L-glutamine (2 mM; Sigma-Aldrich), heparin (100 μg/ml; Sigma-Aldrich) and EC growth supplement (5 μg/ml; Sigma-Aldrich). All cell lines were routinely tested for mycoplasma.

### Freshly isolated endothelial cell culture

All the procedures with the mice (male and female, 6 week old, strain C57/bl6) were performed in agreement with the Institutional Animal Care and Use Committee of the FIRC Institute of Molecular Oncology, in compliance with the guidelines established in the Principles of Laboratory Animal Care (Directive 86/609/EEC), and approved by the Italian Ministry of Health (authorization number 3/2018-PR).

Brain microvascular fragments were processed as described previously (Liebner et al., 2000; Calabria et al., 2006). Capillary fragments were seeded into collagen I (354236, BD Biosciences)-coated wells and cultured in MCDB131 with 20% FBS (Gibco) supplemented with 100 mg/ml heparin (H3149, Sigma-Aldrich) and 5 mg/ml endothelial cell growth supplement (E2759, Sigma-Aldrich). After 3 days of puromycin selection (4 mg/ml; AG-CN2-0078, Adipogen), the cells underwent two rounds of infection with lentiviral vectors for the shRNA-based knockdown of YAP and were cultured until confluence. Then, the cells were starved in MCDB131, plus penicillin/streptomycin (100 U/ml, 0.1 mg/ml), for 8 h, followed by treatment with 5 ng/ml TGFβ for 24 h.

### Cell treatments

For 5 days of TGFβ stimulation, EC were seeded at 3.6×10^4^ cells/cm^2^ density. The day after plating, cells were starved overnight with starving medium [MCDB-131, 1% bovine serum albumin (BSA)], followed by 5 ng/ml TGFβ1 (PeproTech) treatment or vehicle. Fresh TGFβ in starving medium was added every day for 5 consecutive days. For acute TGFβ stimulation, wild-type EC were seeded at a density of 4.2×10^4^ cells/cm^2^ and knockout EC at 5×10^4^ cells/cm^2^. Confluent monolayers of EC were incubated with starving medium overnight, followed by treatment with either 5 ng/ml TGFβ or vehicle in starving medium for the indicated time points.

For LiCl treatment, cells were grown until confluency and then incubated overnight with starving medium containing either 60 mM LiCl (Sigma Aldrich) or 60 mM NaCl (Sigma Aldrich). The day after, cells were stimulated for the indicated intervals with 5 ng/ml TGFβ, dissolved in starving medium together with either 60 mM LiCl or NaCl.

### siRNA transfection

For siRNA transfection, cells were plated at a density of 0.6×10^5^ cells/cm^2^ in complete medium and transfected with either scrambled (SCR) siRNA (ON-TARGET plus Non-targeting pool; GE Healthcare), SMAD3 siRNA (ON-TARGET plus L-040706-00; GE Healthcare) or TEAD1 siRNA (ON-TARGET plus L-048419; GE Healthcare). Transfection was performed with Lipofectamine 2000 (Invitrogen) in accordance with the manufacturer's instructions.

### Lentiviral preparation and infection

Constitutively active YAP lentiviral plasmid (YAP 5SA) was a kind gift from Prof. Stefano Piccolo (Padua University, Italy) (Dupont et al., 2011), wherein a human Flag-YAP 5SA was inserted into a CSII-CMV-MCS-IRES2-Bsd lentiviral backbone plasmid using EcoRI and NotI restriction sites; the empty vector was used as negative control.

The shRNAs for the knock down of TAZ and YAP were as follows: WWTR1 MISSION shRNA plasmid DNA SHCLND-NM_133784 – TRCN0000095953; and YAP1 MISSION shRNA shRNA Plasmid DNA SHCLND-NM_009534 – TRCN0000238432 and TRCN0000095866 (all in a pLKO.1 backbone from Merck KGaA). The scramble shRNA (Addgene, 1864; Sarbassov et al., 2005) was a gift from Davide Sabatini.

The lentiviral particles were produced in HEK 293T cells using a three-plasmid transfection system mediated by Lipofectamine 2000. Twenty-four hours before transfection, 2×106 HEK 293T cells were plated in 57 cm^2^ petri dishes. The following day, cells were transfected with a 3 ml OptiMEM solution containing 4.5 µg psPAX2 (packaging plasmid encoding for Gag, Pol, Rev, and Tat), 1.5 µg of pMD2.G (envelope plasmid encoding for VSV-G), 6 µg of gene transfer vector and 36 µl of Lipofectamine 2000 per Petri dish. This solution was kept for 30 min at room temperature, applied drop by drop on cells and left overnight for transfection. The day after, transfected HEK 293T medium was replaced with DMEM 10% South American FBS and L-glutamine. In parallel, 2×10^6^ of YAP wild-type and knockout cells were seeded in 57 cm^2^ petri dishes. Lentivirus-containing supernatants were collected 48 and 72 h after cell transfection, passed through a 0.45 μm filter and applied to YAP wild-type and knockout cells using polybrene for 24 h. YAP wild-type and knockout infected cells were then grown until confluency using complete culture medium, and then seeded for cell treatments as described previously.

### Gene expression analysis

Total RNA was isolated using an RNeasy Mini kit (Qiagen, Santa Clarita, CA, USA), and 500 ng total RNA was reverse transcribed with random hexamers (High-Capacity cDNA Archive Kits; Applied Biosystems), following the manufacturer's instructions. The cDNAs were amplified using the TaqMan gene expression assay (Applied Biosystems, Foster City, CA, USA) and an ABI/Prism 7900 HT thermocycler. For the quantification of gene expression, the comparative Ct method was used. Briefly, two or three housekeeping genes were analyzed in each experiment. The average of Ct values of the housekeeping genes was calculated and used as reference Ct (Ct-ref). For each gene of interest, we calculated the deltaCt as follows: deltaCt=Ct-gene−Ct-ref. Then, the data were expressed as 2^−deltaCt^ for each sample. Finally, for each experiment, we calculated the average of the 2^−deltaCt^ values of the samples of the control group, and we divided the value of each sample for this average. The resulting values were used for the plot and the statistical analysis (see dedicated section below).

### Western blotting

Western blot analysis was performed according to standard protocols. Confluent monolayers of EC were lysed in boiling Laemmli sample buffer [(2% SDS, 20% glycerol, and 125 mM Tris-HCl (pH 6.8)]. Protein concentration was determined using a BCA Protein Assay kit (Thermo Fisher Scientific). Equal amounts of proteins were loaded on a gel, separated by SDS-PAGE and transferred to a Protran Nitrocellulose Membrane (Whatman). After blocking and incubation with primary and horseradish peroxidase-linked secondary antibodies, specific bindings were detected using a chemiluminescence system (GE Healthcare). Western blot bands have been quantified using optic densitometry software and normalized to the levels of an appropriate housekeeping protein.

### Nuclear-cytoplasmic fractionation

Confluent monolayers of EC were lysed in pre-chilled cytosol buffer [20 mM HEPES (pH 7.9) and 1 mM EDTA (pH 8.0) and protease/phosphatase inhibitors]. After centrifugation, the supernatant was collected (cytosolic fraction) while the pellet was washed three times with cytosol buffer, lysed in cold nuclear buffer [20 mM HEPES (pH 7.9), 1 mM EDTA (pH 8.0), 10% glycerol and 420 mM NaCl and protease/phosphatase inhibitors] and ultracentrifuged for 30 min at 50,000 ***g***. The obtained supernatant was collected as a nuclear fraction.

### Immunoprecipitation

Following overnight starvation and treatment with TGFβ, confluent monolayers of EC were solubilized in cold immunoprecipitation lysis buffer [50 mM Tris-HCl (pH 7.4), 150 mM NaCl, 2 mM CaCl2, 0.1% Triton X-100, 0.1% NP-40 and protease/phosphatase inhibitors] and incubated on ice for 15 min. The protein lysate was then precleared with Protein A or G Sepharose beads (GE Healthcare) for 2 h at +4°C. Subsequently, protein concentration was determined using a BCA Protein Assay kit, and equal amounts of protein were incubated with immune antibodies and captured by protein A or G Sepharose beads overnight at 4°C. As a control, immune antibodies were incubated with immunoprecipitation lysis buffer and protein A or G Sepharose beads overnight at 4°C. The following day, beads were washed several times with immunoprecipitation lysis buffer and boiled in an appropriate volume of Laemmli sample buffer (SB). Immunoprecipitated material was analyzed through standard western blot analysis.

### Immunoprecipitation from nuclear-cytoplasmic fractionation

Cells were lysed with cold subcellular fractionation buffer [250 mM sucrose, 20 mM HEPES (pH 7.4), 10 mM KCl, 1.5 mM MgCl2, 1 mM EDTA, 1 mM EGTA and protease/phosphatase inhibitors]. Cytosolic membranes were disrupted by passing the lysate through a 22 Ga needle, followed by 5 min centrifugation at 720 ***g***. The supernatant was collected as cytosolic fraction, further centrifuged at 10,000 ***g*** for 10 min and immunoprecipitated as described previously. The nuclear pellet was resuspended in cold immunoprecipitation lysis buffer and kept for 1 h at 4°C under constant rotation. After centrifugation at 13,200 rpm (17,000 ***g***) for 20 min, the supernatant containing the nuclear fraction was subjected to immunoprecipitation.

### Immunofluorescence

Cells were fixed with 4% paraformaldehyde (PFA), permeabilized for 10 min with PBS 0.5% Triton X-100, and incubated for 1 h at room temperature in a blocking solution of PBS with 2% BSA and 5% normal donkey serum. Subsequently, samples were incubated with primary antibodies diluted in blocking buffer for 1 h at room temperature, washed with PBS, followed by appropriate secondary antibody incubation for 1 h at room temperature, and mounted with Vectashield with DAPI (Vector Biolabs). Confocal microscopy was performed at room temperature using a confocal microscope (TCS SP2AOBS; Leica) equipped with violet (405-nm laser diode), blue (488 nm; Argon), yellow (561 nm; solid state) and red (633 nm; HeNe) excitation laser lines before processing with ImageJ. Only adjustments of brightness and contrast were used in the preparation of the figures. For comparison purposes, different sample images of the same antigen were acquired under constant acquisition settings. Image acquisition was performed using a 63×/1.4 NA oil immersion objective (HCX PL APO 63× Lbd BL, Leica) with spectral detection bands, and scanning modalities were optimized for removal of channel crosstalk. Confocal software (Leica) and ImageJ version 1.33 were used for data analysis. Quantification of nuclear accumulation was performed by measuring the intensity of SMAD staining with ImageJ, using DAPI nuclear staining as a region of interest to identify cell nuclei.

### Wound healing assay

Cells were seeded in a six-well plate in complete medium and cultured until a uniform monolayer had formed. After overnight starvation, the cell monolayer was scratched with a pipette tip and carefully washed with 1× PBS to remove floating cells and create a cell-free wound area. The closure of the wound was monitored by time-lapse microscopy. An Olympus ScanR inverted microscope with a 10× objective was used to take pictures every 5-10 min over a 24 h period (as indicated in the figure legends). The assay was performed in complete culture medium using an environmental microscope incubator set to 37°C and 5% CO_2_ perfusion.

### Proliferation assay

Cells were plated in 200 μl of culture medium at a density of 1000 cells/well in 96-well plates. The following day (d=0), a 96-well plate was fixed and used as a cell growth starting point. Cell growth was subsequently calculated at days 1, 2, 3 and 4 after the starting point. The medium was removed and cells were fixed by adding 100 μl of a solution containing 0.1% Crystal Violet in 20% methanol. After being shaken (200 cycles/min) for 20 min at room temperature, plates were washed five times by submersion in deionised water and air dried for at least 24 h. Bound dye was solubilised by adding 100 μl of 10% acetic acid and shaking the plates for 5 min at room temperature. The optical density of the dye extracts was measured directly in plates using a Microplate Reader at a wavelength of 590 nm.

### Transcription factor binding site analysis

The identification of putative SMAD binding sequences on genomic DNA was performed using MatInspector (Genomatix), which predicts the transcription factor binding sites by using a large library of weight matrices. Using RSAT (http://rsat.sb-roscoff.fr/), we retrieved a sequence spanning from 5000 bp upstream and 1000 bp downstream of the transcription start sites of *Fn1*, *Serpine1* and *Snai1* genes.

### ChIP qPCR

ChIP qPCR assays were performed as described previously (Nakae et al., 2003). Briefly, cells were starved overnight and crosslinked with 1% formaldehyde for 10 min at room temperature. Glycine (125 mM) was then added for 5 min at room temperature to inactivate formaldehyde. After two washes with ice-cold PBS EC were lysed by scraping on ice-cold SDS buffer [100 mM NaCl, 50 mM Tris HCl (pH 8.1), 5 mM EDTA, 0.2% NaN3 and 0.5% SDS]. The lysate was then collected and centrifuged at 1300 ***g*** for 5 min at 4°C. After removal of the supernatant, the pellet was resuspended with immunoprecipitation buffer {1 volume of SDS buffer plus 0.5 volume of Triton dilution buffer [100 mM NaCl, 100 mM Tris HCl (pH 8.6), 5 mM EDTA, 0.2% NaN3, Triton X-100 5%]}. Sample sonication in microTUBE (Covaris) was performed after 10 min of incubation using a Covaris S220 ultrasonicator according to the following conditions: peak incident power, 175.0 watts; duty factor 10%; and 200 cycles/burst. Sonicated chromatin was loaded on a 1% agarose gel to evaluate the size of the sonicated chromatin fragments. DNA fragments [0.5 mg (for YAP) or 0.3 mg (for SMAD3)] with an average size of 500 bp were incubated with either 8 µg of YAP (NB110-58358) or 1.5 µg of SMAD3 (cs#9523) directed antibodies or rabbit IgG control overnight at 4°C in the presence of protein G-covered magnetic beads (Life Technologies). The following day, beads were recovered and washed three times with mixed micelle washing buffer [150 mM NaCl, 20 mM TrisHCl (pH 8.1), 5 mM EDTA, 5.2% sucrose w/v, 0.02% NaN_3_, 1% Triton X-100 and 0.2% SDS], 500 buffer (0.1% deoxycholic acid w/v, 500 mM NaCl, 25 mM HEPES (pH 7.5), 1 mM EDTA, 0.02% NaN_3_, 1% Triton X-10) and LiCl detergent washing buffer [0.5% deoxycholic acid (w/v), LiCl 250 mM, 1 mM EDTA, 0.5% NP-40 (v/v), 0.02% NaN_3_ and 10 mM Tris HCl (pH 8.0)]. Proteins/DNA complexes were detached from beads by heating the samples at 65°C for 10 min. De-crosslinking was performed at 65°C overnight. DNA was precipitated and purified using phenol/chloroform and amplified by qPCR using oligonucleotides flanking the assayed promoter regions (listed below). Primers were designed using Primer3 software and were always tested in advance to avoid ‘auto-amplification’ due to self-complementarity. qPCR reactions were carried out by diluting DNA in the presence of specific primers (0.4 μM each) to a final volume of 25 μl in SYBR Green Reaction Mix (Perkin Elmer). S.D.S 2.2.1 software was used to convert qPCR curves to Ct values. For each region, the mean of the Cts of the inputs was calculated and subtracted from the Ct values of the immune samples (ΔCt). Then, the percentage of enrichment of input for the immune samples was obtained as 2^−ΔCt^ and multiplied by the percentage of input taken during the experiment. The same calculation was performed for the non-immune (IgG control) immunoprecipitated samples. The following primers were used for the qPCR:

Fn1 gene (position: −0.3 kb), forward, 5′-GTAAGCCTTACCACCCCAGG; reverse, 3′-GGGATGGGAAACGGCTGTAA-5′.

### Antibodies and reagents

The following reagents were used: recombinant human TGF-β1 (100-21C, PeproTech) and recombinant BMP6 (507-BP-020, R&D Systems). For western blot, immunofluorescence, immunoprecipitation and ChIP qPCR, the following primary antibodies were used: phospho-SMAD3 (s423/s425) rabbit IgG (9520, Cell Signaling Technology; western blot, 1:1000), phosphor-SMAD3 (s204) rabbit IgG (Ab63402, Abcam; western blot, 1:1000), SMAD3 rabbit IgG (9523, Cell Signaling Technology; western blot, ChIP qPCR), SMAD3 mouse IgG (sc-101154, Santa Cruz Biotechnology; western blot, 1:1000), SMAD3 mouse IgG (MA5-15663, Thermo Fisher Scientific; western blot, 1:1000), phospo-SMAD2 rabbit IgG (3108, Cell Signaling Technology; western blot, 1:1000), SMAD2 rabbit IgG (ab33875, Abcam; western blot, 1:1000, and immunofluorescence, 1:100), SMAD4 goat IgG (sc-1909, Santa Cruz Biotechnology; western blot, immunofluorescence, 1:100, and immunofluorescence, 1:200), YAP mouse IgG (sc-101199, Santa Cruz Biotechnology; western blot, 1:2000, and immunofluorescence, 1:200), YAP mouse IgG (sc-271134, Santa Cruz Biotechnology; immunofluorescence, 1:100, and immunoprecipitation, 1:200), YAP rabbit IgG (sc-15407, Santa Cruz Biotechnology; western blot, 1:1000), YAP rabbit IgG (110-58358, Novus Biologicals, ChIP qPCR), VE-Cadherin goat IgG (sc-6458, Santa Cruz Biotechnology; immunofluorescence, 1:200), FN1 rabbit IgG (ab23750, Abcam; western blot, 1:2000), Snai1 goat IgG (sc-10432, Santa Cruz biotechnology; western blot, 1:1000), tubulin mouse IgG (T9026, Sigma-Aldrich; western blot, 1:5000), vinculin mouse IgG (V9264, Sigma-Aldrich; western blot, 1:5000), lamin B goat IgG (sc-6216, Santa Cruz Biotechnology; western blot, 1:1000), p120 mouse IgG (610133, BD Biosciences; immunofluorescence, 1:100), plakoglobin mouse IgG (610253, BD Biosciences; immunofluorescence, 1:100), β-catenin mouse IgG (610153, BD Biosciences; immunofluorescence, 1:200), JAM-A goat (AF1077, R&D Systems; western blot, 1:1000), claudin5 mouse IgG (352588, Thermo Fisher Scientific; immunofluorescence, 1:100), claudin5 mouse IgG (352500, Thermo Fisher Scientific; western blot, 1:100).

### Statistical analysis

Statistical analysis was performed using GraphPad Prism 8. Datasets were first tested for normal distribution using the Shapiro–Wilk test. The data showing normal distribution were analyzed using parametric tests, i.e. unpaired Student's *t*-test or one-way ANOVA followed by Fisher's least significant difference (LSD) post-hoc test. Non-parametric data were analyzed using Mann–Whitney's or Kruskal–Wallis's tests. For each plot, the statistical test applied is specified in the corresponding legend.

## Supplementary Material

Supplementary information

Reviewer comments
