# Supplementary material for: A dual role of YAP in driving TGFβ-mediated endothelial-to-mesenchymal transition
Source: J Cell Sci. 2021 Aug 2;134(15):jcs251371. doi: 10.1242/jcs.251371 (PMC8353525; doi:10.1242/jcs.251371)
Supplement: Supplementary information [file joces-134-251371-s1.pdf]

Figure S1

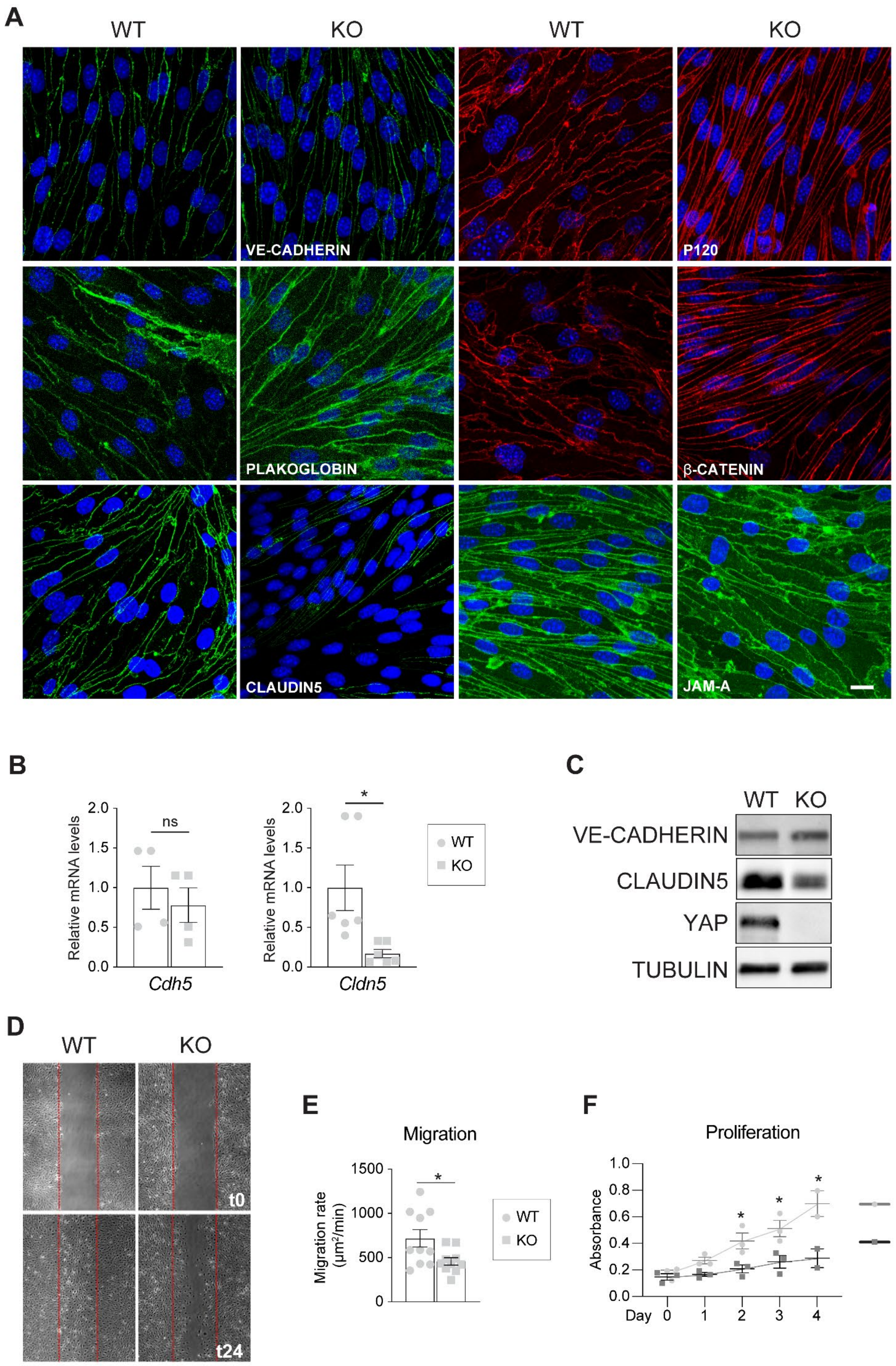

Journal of Cell Science • Supplementary information

Fig.S1. Immortalized cells retain their endothelial nature, the absence of YAP impairs proliferation and migration properties.

A) Representative IF staining of VE-CADHERIN, p120, PLAKOGLOBIN, β-CATENIN, CLAUDIN5 and JAM-A in YAP WT and KO cells; nuclei are counterstained with DAPI. Scale bars = 10 μm.

B) RT-qPCR analysis of the endothelial genes *Cdh5* and *Cldn5* mRNA expression levels in YAP WT and KO cells. Samples are normalized to WT cells. Data are mean ± SEM of n=4-6 independent samples. *$p<0.005$ (Mann-Whitney's test).

C) WB analysis for expression of VE-CADHERIN, CLAUDIN5 and YAP in YAP WT and KO EC. TUBULIN was used as a loading control.

D-E) Wound healing assay to assess migration rate in WT and KO cells. D) Representative images of WT and KO cells after 0 (t0) and 24 hours (t24) after the scratch. E) Quantification of the migration rate of WT and KO cells in wound healing assay; Data are mean ± SEM of n=10 independent samples and are expressed as μm/minute. *$p<0.05$ (Unpaired t test).

F) Proliferation assay on WT and KO performed for 4 days. Data are mean ± SEM of n=3 independent samples. $p<0.001$ among time points and between genotypes (two-ways ANOVA),*$p<0.005$ vs WT of the same time point (Fisher's LSD post hoc test).

Journal of Cell Science • Supplementary information

Figure S2

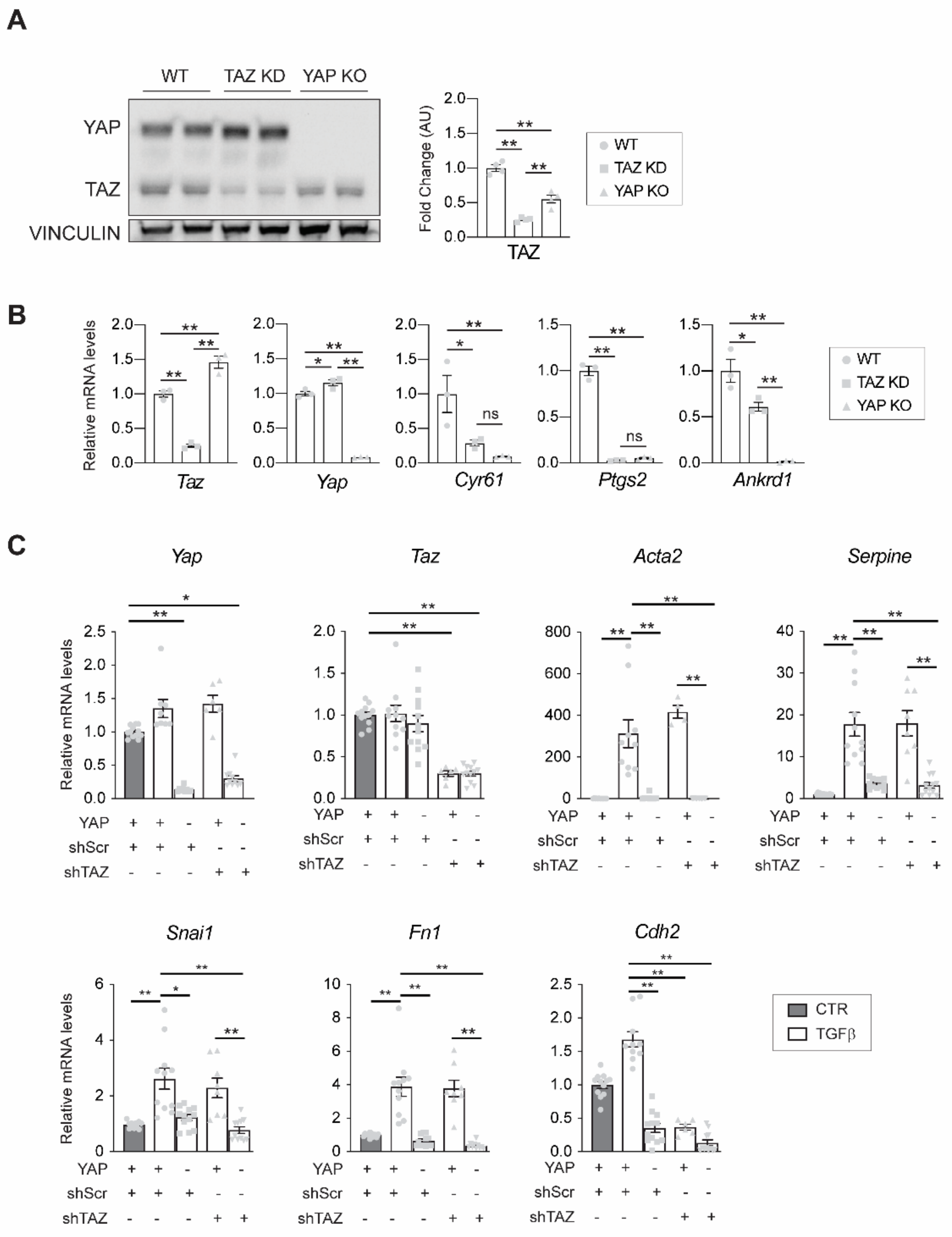

Fig. S2. TAZ does not regulate TGFβ-mediated EndMT.

A) WB (left panel) and relative quantification (right panel) of TAZ and YAP levels in WT, TAZ KD and YAP KO EC. Vinculin was used as a loading control. Data in the plots are mean ± SEM normalized to WT cells; $p<0.0001$ among groups (one-way ANOVA); $^{**}p<0.001$ (Fisher's LSD post hoc test).

Journal of Cell Science • Supplementary information

B) RT-qPCR analysis of *Taz, Yap, Cyr61, Ptgs2* and *Ankrd1* mRNA expression levels in YAP WT and KO cells that were infected with either shSCR or shTAZ lentiviral vectors. Samples are normalized to WT shSCR cells. Data are mean ± SEM of n=3 independent samples. $p<0.02$ among groups (one-way ANOVA), *$p<0.05$, **$p<0.01$ (Fisher's LSD post hoc test).

C) RT-qPCR analysis of *Yap, Taz* and the EndMT markers *Acta2*, *Serpine1*, *Snai1*, *Fn1*, and *Cdh2* mRNA expression levels in YAP WT and KO cells that were infected with either shSCR or shTAZ lentiviral vectors and treated with 5 ng/ml TGFβ for 5 days. Samples are normalized to WT untreated cells. Data are mean ± SEM of n=8-12 independent samples. $p<0.0001$ among groups (one-way ANOVA), *$p<0.5$, **$p<0.01$ (Fisher's post hoc test).

Journal of Cell Science • Supplementary information

Figure S3

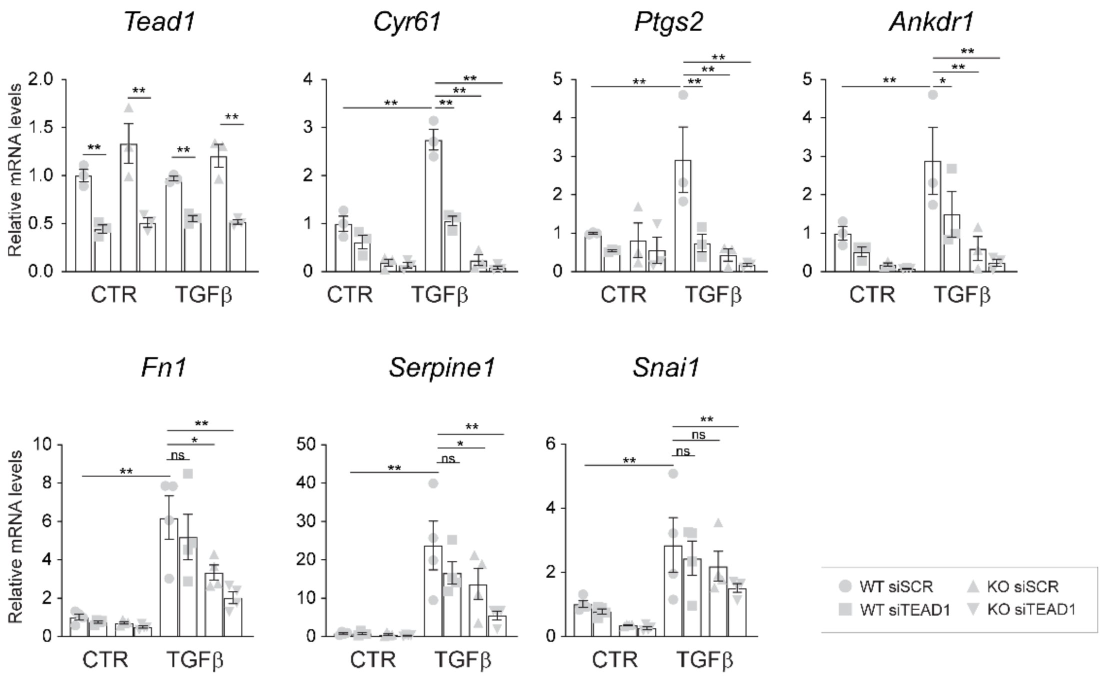

Fig. S3. TEAD is not involved in TGFβ-mediated EndMT.

RT-qPCR analysis of *Tead1, Cyr61, Ptgs2, Ankrd1* and the EndMT markers *Fn1, Serpine1 and Snai1* mRNA expression levels in YAP WT and KO cells that were transfected with either siSCR or siTEAD1 and treated with 5 ng/ml TGFβ for 24h hours. Samples are normalized to WT untreated cells. Data are mean ± SEM of n=3-4 independent samples. p<0.05 among groups (one-way ANOVA), *p<0.5, **p<0.01 (Fisher's post hoc test).

Journal of Cell Science • Supplementary information

Figure S4

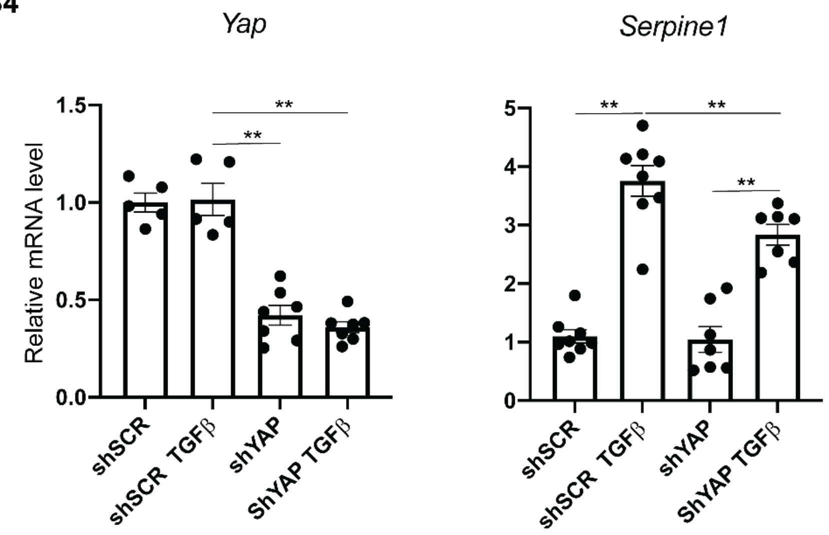

Fig. S4. YAP regulates TGFβ-induced transcription program in primary EC. RT-qPCR analysis of Yap and Serpine1 mRNA expression levels in freshly isolated primary endothelial cells that were infected with either shSCR or shYAP lentiviral vectors and treated with 5 ng/ml TGFβ for 24h hours. Samples are normalized to WT untreated cells. Data are mean ± SEM of n=8 biological replicates. $p<0.0001$ among groups (one-way ANOVA), *$p<0.5$, **$p<0.01$ (Fisher's post hoc test).

Journal of Cell Science • Supplementary information

Figure S5

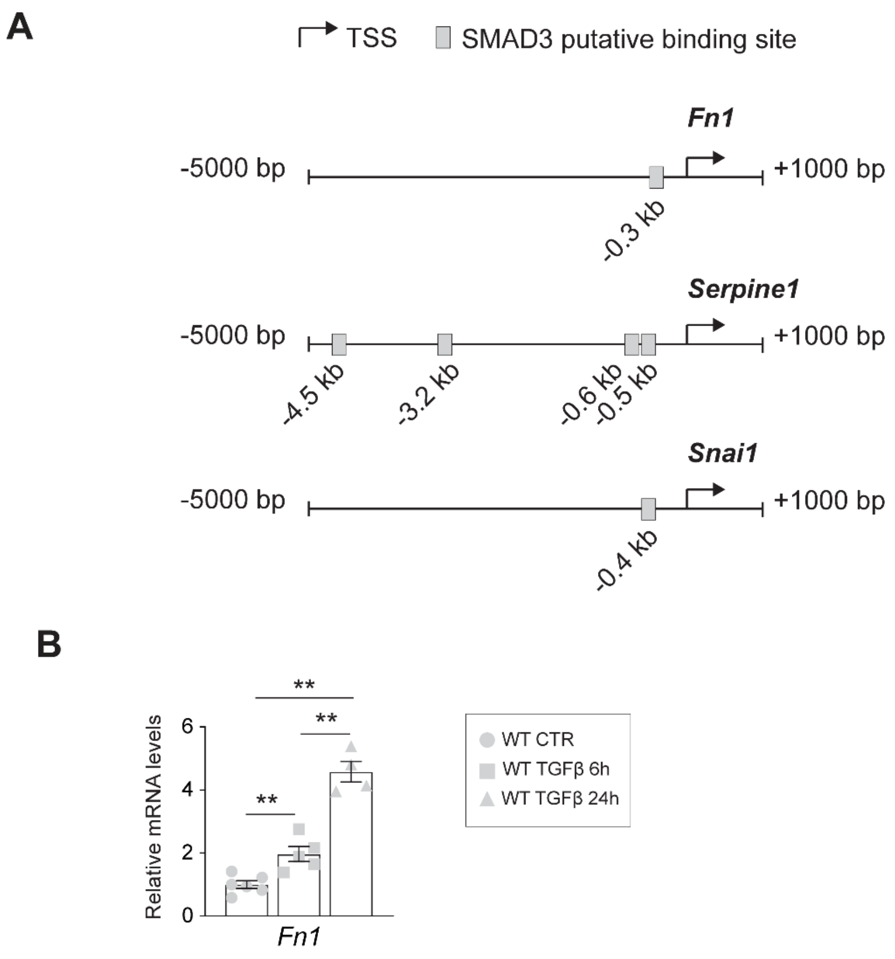

Fig. S5. SMAD3 binds the promoter of Fn1 in a time dependent manner.

A) Schematic illustration of the promoter regions spanning - 5.0 KB to + 1.0 KB around the transcription start site (TSS) of *Serpine1*, *Fn1*, and *Snai1*. Boxes represent SMAD3 putative binding site. Analysis has been performed retrieving the sequence from RSAT and then searching for putative binding sites using MatInspector software.

B) RT-qPCR analysis of Fn1 expression in a TGFβ treatment time-course. Data are mean of n=3 independent experiments ± SEM. $p<0.0001$ among groups (one-way ANOVA), **$p<0.01$ (Fisher's LSD post hoc test).

Journal of Cell Science • Supplementary information
