# Supplementary material for: A dual role of YAP in driving TGFβ-mediated endothelial-to-mesenchymal transition
Source: J Cell Sci. 2021 Aug 2;134(15):jcs251371. doi: 10.1242/jcs.251371 (PMC8353525; doi:10.1242/jcs.251371)
Supplement: Reviewer comments [file joces-134-251371_review_history.pdf]

A dual role of YAP in driving TGFβ-mediated endothelial-to-mesenchymal transition

Cecilia Savorani, Matteo Malinverno, Roberta Seccia, Claudio Maderna, Monica Giannotta, Linda Terreran, Eleonora Mastrapasqua, Stefano Campaner, Elisabetta Dejana and Costanza Giampietro
DOI: 10.1242/jcs.251371

Editor: Kathleen Green

Review timeline

Original submission:	7 July 2020
Editorial decision:	17 August 2020
First revision received:	6 April 2021
Editorial decision:	19 May 2021
Second revision received:	11 June 2021
Accepted:	23 June 2021

Original submission

First decision letter

MS ID#: JOCES/2020/251371

MS TITLE: A Dual Role of YAP in Driving TGFβ-mediated EndMT

AUTHORS: CECILIA SAVORANI, MATTEO MALINVERNO, ROBERTA SECCIA, CLAUDIO MADERNA, MONICA GIANNOTTA, STEFANO CAMPANER, ELISABETTA DEJANA, and Costanza Giampietro
ARTICLE TYPE: Research Article

We have now reached a decision on the above manuscript.

To see the reviewers' reports and a copy of this decision letter, please go to: https://submit-jcs.biologists.org and click on the 'Manuscripts with Decisions' queue in the Author Area. (Corresponding author only has access to reviews.)

As you will see, the reviewers are generally positive about the pathway studied in the paper and its potential interest to the field. However, they raise a number of substantial criticisms that prevent me from accepting the paper at this stage. A central issue agreed upon by all the referees (including referee #1 in post-review discussion) is that that the findings are inherently limited by the use of an immortalized line, and would be strengthened by validating in a primary cell lines. There was also concern expressed about the limited characterization of the cells and of EndMT.

If you can address these and other detailed concerns, I would be pleased to see a revised manuscript. We would then return it to the reviewers.

We are aware that access the lab to undertake experimental revisions has been limited over the past several months and may still provide a challenge with respect to addressing all the criticisms. However, in my opinion and the opinion of all of the referees, several critical experimental issues do exist that will required additional time at the bench. If it would be helpful, send us a point-by-point response indicating where you are able to address concerns raised (either experimentally or by changes to the text) and where you will not be able to do so within the normal timeframe of a revision. We will then provide further guidance. Please also note that we are happy to extend

© 2021. Published by The Company of Biologists under the terms of the Creative Commons Attribution License (https://creativecommons.org/licenses/by/4.0/).

revision timeframes as necessary. Another option in this case would be to submit to another journal so as not to delay publication.

Please ensure that you clearly highlight all changes made in the revised manuscript. Please avoid using 'Tracked changes' in Word files as these are lost in PDF conversion.

I should be grateful if you would also provide a point-by-point response detailing how you have dealt with the points raised by the reviewers in the 'Response to Reviewers' box. Please attend to all of the reviewers' comments. If you do not agree with any of their criticisms or suggestions please explain clearly why this is so.

Reviewer 1

Advance summary and potential significance to field

In this work, Savorani, Malinverno, and their co-authors demonstrate YAP-Smad3 complex formation and cross-talk yielding synergistic induction of TGF-beta responsive genes in immortalized lung-derived endothelial cells. YAP has been shown to be of importance to EndMT in vivo and in vitro previously (Zhang et al.) via YAP-SMAD3 transcriptional interactions. The Varelas lab and others have also demonstrated the existence of TGF-beta - YAP/TAZ crosstalk via Smad3 interactions in epithelial and stem cell systems. These authors validate YAP-Smad3 complex formation and transcriptional synergy, but also demonstrate that YAP-SMAD3 binding prevents GSK-mediated SMAD3 phosphorylation and degradation to enhance SMAD3 nuclear localization and gene transcription. The authors suggest Hippo/YAP pathway targeting may offer an alternative approach to targeting EndMT (and presumably other TGF-beta dependent processes) in endothelial cells.

Comments for the author

The authors are to be commended for their methodical approach to examining and mechanistically characterizing the suppressed TGF-beta response found in their YAP KO immortalized cell line. Their experimental process is straightforward and their hypotheses very reasonable based on established literature in the field. Several issues will need to be further addressed, however, before this data is ready for publication.

Major Comments:
1) The authors refer frequently in their abstract and introduction to the idea that they are interrogating EndMT, which, as they summarize involves "acquiring mesenchymal features such a spindle-shape morphology proliferative, invasive, and migratory properties, weakening of cell-cell junctions, loss of endothelial-specific markers, gain of mesenchymal markers and adjustment of cytoskeletal organization." Their phenotype as presented however, demonstrates only the induction of TGF-beta responsive genes, albeit some of them that are mesenchymal markers (aSMA, Id1) and known early TFs involved in EndMT (Snail1, Serpine1). I have examined the referenced works from their group, but these generally demonstrate EndMT phenotypes in CCM KO cells (in contrast to WT cells with much less robust phenotypes). The paper would be stronger with experimental work more clearly demonstrating EndMT phenotypes (and EndMT inhibition with YAP KO) in their experimental system; in the absence of this the authors should consider rephrasing their work to discuss "early gene markers of EndMT" or "EndMT early transcriptional program" and rephrase their abstract and introduction more carefully to indicate their focus on this aspect of the phenotype only.

2) In multiple figures (1B, 1C, 1D, 2A, 2B, 2C, 3A, 4B, 5B, 5E, 6B, 6C, 6D S3B) the authors appear to have used one-way ANOVA and ANOVA post-tests on normalized data. As ANOVA is an analysis of variance, including samples with no variance due to normalization artificially enhances statistical power and is inappropriate. The approach of figure S1B is better, where all the individual values are maintained and then normalized, allowing variance in the control group. I don't entirely agree with parametric testing (ANOVA rather than Kruskal-Wallis) in this setting either; however, this is commonly done, and I will defer it to the editor's/journal's discretion.

3) Similarly, the qPCR data that makes up much of the data in the paper should be presented and analyzed in log scale, as this is the scale on which the measurements (deltaCT/delta-deltaCT

© 2021. Published by The Company of Biologists under the terms of the Creative Commons Attribution License (https://creativecommons.org/licenses/by/4.0/).

values) are performed. It is clear this has not been done based on the SEM error bars; curiously the manuscript does not at present contain a qPCR methods section so that I know what has been done in terms of analysis and seems to perform the statistics differently in different figures (as noted above). I am suspicious that considerable baseline downregulation of TGF-beta responsive genes in YAP KO cells and significant induction of those genes (from a low baseline) by TGF-beta is being obscured by the data as currently presented (in linear scale). For example, I suspect there is a statistically significant upregulation of Acta2, Fn1,and Serpine1 with TGF-beta induction (Figure 1a) when analyzed appropriately in log scale.

4) The authors make a strong claim that their phenotype is YAP-specific, with no contribution from TAZ. However, they are using YAP KO cells, while their shRNA for TAZ demonstrates only about 60% reduction. I am quite concerned that Serpine1 and Acta2, genes that have known TEAD binding domains [Ota et al., Development 135, 4059-4069 (2008)] are not impacted by TAZ reduction at all suggesting that either the knockdown is insufficient or TAZ is not driving TEAD transcriptional activation at all in these cells. What is the relative expression of Yap and TAZ in the cells (by CT value?). Is it possible that TAZ is just not very highly expressed? At a minimum, the authors' strong claims would be enhanced by demonstrating a TAZ-specific phenotype that is impacted (another canonical TEAD gene such as CTGF, CYR61, ANKRD1, etc. for example, or a decrease in a TEAD reporter assay). An even stronger approach would be to try overexpressing TAZ4SA in their knockout cells and show that this does not rescue their phenotype. Without additional data, I think they can only state that 2.5-fold reduction in TAZ levels did not have a strong impact on their phenotype in this cell line.

Minor Comments:
1) Page 6 - I am not sure the relevance of the Qin paper to your manuscript other than being more recent - it mostly makes me wonder whether the authors should have looked at Smad7 themselves. It might be better for it to be included in the list of references and a more general discussion that YAP and TGFb/Smad3 interactions are well-established in other cell systems.

2) The current text doesn't seem to grapple much with the reduction in Smad3 mRNA (demonstrated in figure 2B and 6B) in the YAP KO line. The data showing that reintroduction or increase in nuclear YAP prevents degradation (via GSK) [fig 2C, 5A-C] is solid, but the authors do not address or speculate about why YAP KO cells have lower transcriptional SMAD3 activity, or whether this impacts interpretation of any of their data (in particular Figure 3A).

3) Page 11 - The suggestion that YAP phosphorylation stabilization means there is no nuclear accumulation of YAP is not really accurate (it does, however suggest no activation of canonical Hippo pathway signaling). There has been significant evidence that mechanical cues, for example, do not require changes in YAP phosphorylation to induce nuclear YAP shuttling (see Elosegui-Artola et al., Cell Volume 171, Issue 6, 30 November 2017, Pages 1397-1410.e14). The nuclear/cytoplasmic fractionation is much more convincing that there are not strong changes in YAP localization with TGF-beta stimulation. A small change of phrasing would be helpful.

4) There are no immunofluorescence methods or reference to such methods. The YAP staining is fairly unconvincing - a positive control for nuclear localization (for example, the YAP5SA cells) would be helpful.

5) In figure 5E, why is Serpine1 not stimulated in the NaCl condition? Why was the immunofluorescence performed at 2h while the qPCR performed at 24hours? If it is known by western, N/C fractionation, or IF that the nuclear (or total) accumulation of SMAD3 persists between 2 and 24h of LiCl treatment in the KO cells it should be shown or at least stated.

6) Figure 6A should include either the IgG controls for each set of cells or the Y-axis needs to labeled properly to explain the normalization performed based on the methods. It would be preferable to show the IgG controls as in Zhang et al (2014). Experimental triplicates should not have error bars; if 3 independent experiments were done, then the data should be pooled and shown.

7) There is some confusing language in the text surrounding YAP as a "co-transcriptional factor" for SMAD3 and "binding" to the Smad3 promoter. YAP is a transcriptional co-regulator (transcriptional

© 2021. Published by The Company of Biologists under the terms of the Creative Commons Attribution License (https://creativecommons.org/licenses/by/4.0/).

cofactor is acceptable language as well), and does not bind DNA directly. The authors have shown YAP-SMAD3 binding directly and indirectly throughout this paper, and their data suggests it allows for increased nuclear accumulation and stabilization of SMAD3. Their CHIP assay demonstrates YAP and Smad3 are both present at the Fn1 promoter site. It would be helpful to adjust the text for clarity.

8) The authors at times appear to overstate the requirement for both YAP and SMAD3 for transcriptional induction of their genes of interest. The data show fair induction of Fn1 and Serpine1 in the SMAD3+/YAP-/TGFb, YAP+/siSMAD3/TGFb and unstimulated YAP5SA conditions in figure 6, suggesting a co-operative additive, or possibly synergistic effect on the TGF-beta mediated transcription of these genes (neither is 100% essential). I would recommend looking over the text generally to make sure that statements about things that are "essential" or "required" are fully backed up by the data.

9) The authors have not ruled out the possibility that YAP is directing TEAD-mediated transcription of the TGF-beta responsive genes as an important factor rather than enhancing SMAD3 transcription directly (as opposed to indirectly through nuclear sequestration or stabilization). Certainly Hiemer et al. would suggest TEAD binding is important for Serpine1 transcription, for example. A brief discussion of this point (or an experiment to rule out TEAD-mediated effects, such as using YAP5SA-S94A expressing cells) is required.

10) The authors should comment on the fact that using an immortalized cell line to assay pro-proliferative phenotypes (EndMT) involving a known oncogene (YAP) has some inherent limitations.

Reviewer 2

Advance summary and potential significance to field

The manuscript is a detailed biochemical study of the role of YAP in SMAD3-dependent signalling and EndoMT. The findings are novel and relevant to a field which has been growing over the last few years because of the potential implications of EndoMT in many human pathologies.

Comments for the author

This is an overall well conducted study, with some limitations discussed below.

1. Immortalised mouse lung EC are used in this study. The Authors should provide basic EC characterisation with images of the cells by IF and PRC to show how much of the endothelial phenotypes is retained. This is important in general, but more so here given that the claim to novelty is the effect on the endothelial lineage; the pathways under investigation have already been described other lineages.

2. To exclude possible bias from immortalised EC lines, another endothelial model of YAP ko, perhaps by siRNA, should be used to validate some of the key findings in HUVEC or another human primary endothelial cell

3. Fig1D: expression levels of Id1 and Serpine1, are used as readout of ALK1/SMAD1 and ALK5/SMAD3 signalling, respectively. Based on the regulation of these, they conclude "….. that TGFβ downstream signaling is impaired in absence of YAP and that YAP is specifically required for SMAD3 but not SMAD1 signaling in EC." A few more targets of these pathways should be measured to support this statement.

4. Page9 and Fig 2A: TGFb induced P of SMAD1 and 3, and YAP regulation of SMADs expression. Statistical analysis should be carried out between WT and KO because it looks like the KO cells have nearly double the level of P SMAD3 in response to TGFb. The Authors should comment on this finding: why is Yap KO increasing TGFb-dependent SMAD1 and 3 Phosphorylation. Also, the text on page 9 only focuses on regulation of SMAD3, but very similar effects are seen with SMAD1. This is in contrast with what stated above (Fig 1D). Does TNFb significantly increase P of SMAD1 but not

© 2021. Published by The Company of Biologists under the terms of the Creative Commons Attribution License (https://creativecommons.org/licenses/by/4.0/).

SMAD3? Stats are missing. For the rest of the study, SMAD1 is ignored; but based on this initial finding, it seems that SMAD1 may be playing a role too. This needs more detailed investigation.

5. Fig 2D Page 10: The Authors state "SMAD3 phosphorylation occurred at comparable levels between the two cell lines, suggesting that YAP did not influence SMAD3 C-term phosphorylation". But the figure shows statistical significance between WT and KO - that's confusing.

6. An important aspect of this story seems to be the regulation of SMAD3 expression by YAP. Can the Authors speculate about the mechanism and the implications on their findings?

Other comments:

1. R-SMADs are introduced on page 8 without any explanation of the abbreviation.

2. General comment: in the Results, there is lengthy background information with references. The Result section should be revised and perhaps figures reorganised to allow for a more linear narrative. Some of the discussion on the relevant literature should be moved to introduction or discussion (example: the first 8 lines of the results)

3. All bar graphs: The symbols are very small and very similar to each other, making it difficult to discriminate between them. Please use larger symbols, and more clearly different between each other.

4. Figures are not described with enough detail. Example: Fig 6B. The figure has 3 different graphs, each with 8 conditions, with very small legends.
The reader has to struggle through it all without much explanation. The Authors should check all their figure description in the text and improve where necessary.

5. Fig S1 and S2 shows that SMAD3 binds YAP. This is new data important for this story, and should be in the main paper.

6. Fig 4E: TGFb treatment and YAP/SMAD3, showing a representative image of 3. Quantification of all experiments should be shown, given that the effect seems pretty small

7. Fig 5A shows that SMAD3 Phosphorylation upon TGFb timecourse is higher in YAP ko cells. They already showed that in Fig 2A, without the timecourse: the data should be reorganised for clarity and consistency

8. Fig 6B shows that ko of SMAD3 reduces levels of the two target genes analysed in WT but not YAP ko cells. The graph shows great variability in the Fn1 samples for the KO + siControls. The conclusions would be more reliable with a few more datapoints. On this figure, the text states: "Data showed that SMAD3 is necessary for inducing both Fn1 and Serpine1 expression upon TGFβ stimulation, further indicating that SMAD3 is important for driving EndMT gene transcription." But we knew this already; this is not the conclusion from this figure, which aims to investigate the possible synergy between SMAD3 and YAP.

9. The shortcut "ChIP assay" is used here for ChIP qPCR, which is the correct term.

<u>Reviewer 3</u>

Advance summary and potential significance to field

The manuscript by Savorani et al., focuses on the role of the co-transcriptional regulator YAP in TGFbeta-induced endothelial to mesenchymal transition (EndMT). Given the role of YAP signaling in orchestrating TGFbeta signaling in multiple cell types, the authors assessed weather loss of YAP in endothelial cells (ECs) blocked EndMT induced by TGFbeta. Using an immortalized endothelial cell line derived from mouse lungs the authors employed a series of biochemical approaches and found that YAP plays a role in SMAD3 protein stability and consequently TGFbeta-induced EndMT. YAP

© 2021. Published by The Company of Biologists under the terms of the Creative Commons Attribution License (https://creativecommons.org/licenses/by/4.0/).

prevented GSK3beta-promoted SMAD3 phosphorylation and degradation. Loss of YAP favored SMAD3 degradation and inhibited TGF-beta signaling.
Overall the study is well presented and results are adequately discussed. The novelty of the study, however, is compromised by the existing literature reporting YAP as a SMADs cofactor that plays a role in TGF-beta signaling in multiple cell types.

Comments for the author

Given the emerging role for YAP in regulating sprouting angiogenesis, the authors should elaborate on how their findings reconciliate with YAP angiogenic functions. For instance, does VEGF signaling interfere with YAP-promoted EndMT in presence of TGFbeta? Can the author speculate on how YAP controls endothelial migration in the contest of EndMT and angiogenesis?

EndMT is associated with the downregulation of junctional proteins, including VE-cadherin, to facilitate endothelial dissociation and mesenchymal differentiation. Does YAP compromise vascular integrity and cellular junctions? The authors should also show endothelial marker staining to ensure cell maintained their endothelial identify following immortalization. Junctional protein staining would also help to validate endothelial integrity before treatments.

My major concern in this study is that the authors do not use primary endothelial cells to demonstrate or corroborate their major findings. The use of an immortalized cell line to evaluate EndMT should be accompanied by further experiments using primary endothelial cells as a model. Given the contribution of EndMT is multiple human disorders, the use of human endothelial cells would strengthen the findings of the study and provide insights on potential therapeutic implications for human disease. Key findings of the study should be reproduced in primary cells.

First revision

Author response to reviewers' comments

RESPONSE TO REVIEWERS

Reviewer 1 Advance Summary and Potential Significance to Field: In this work, Savorani, Malinverno, and their co-authors demonstrate YAP-Smad3 complex formation and cross-talk yielding synergistic induction of TGF-beta responsive genes in immortalized lung-derived endothelial cells. YAP has been shown to be of importance to EndMT in vivo and in vitro previously (Zhang et al.) via YAP-SMAD3 transcriptional interactions. The Varelas lab and others have also demonstrated the existence of TGF-beta - YAP/TAZ crosstalk via Smad3 interactions in epithelial and stem cell systems. These authors validate YAP- Smad3 complex formation and transcriptional synergy, but also demonstrate that YAP-SMAD3 binding prevents GSK-mediated SMAD3 phosphorylation and degradation to enhance SMAD3 nuclear localization and gene transcription. The authors suggest Hippo/YAP pathway targeting may offer an alternative approach to targeting EndMT (and presumably other TGF-beta dependent processes) in endothelial cells.

Reviewer 1
Comments for the Author: The authors are to be commended for their methodical approach to examining and mechanistically characterizing the suppressed TGF-beta response found in their YAP KO immortalized cell line. Their experimental process is straightforward and their hypotheses very reasonable based on established literature in the field. Several issues will need to be further addressed, however, before this data is ready for publication.
We thank the reviewer for the careful reading of our manuscript.

Major Comments:

© 2021. Published by The Company of Biologists under the terms of the Creative Commons Attribution License (https://creativecommons.org/licenses/by/4.0/).

1) The authors refer frequently in their abstract and introduction to the idea that they are interrogating EndMT, which, as they summarize, involves "acquiring mesenchymal features such a spindle-shape morphology, proliferative, invasive, and migratory properties, weakening of cell-cell junctions, loss of endothelial-specific markers, gain of mesenchymal markers, and adjustment of cytoskeletal organization." Their phenotype as presented, however, demonstrates only the induction of TGF-beta responsive genes, albeit some of them that are mesenchymal markers (aSMA, Id1) and known early TFs involved in EndMT (Snail1, Serpine1). I have examined the referenced works from their group, but these generally demonstrate EndMT phenotypes in CCM KO cells (in contrast to WT cells with much less robust phenotypes). The paper would be stronger with experimental work more clearly demonstrating EndMT phenotypes (and EndMT inhibition with YAP KO) in their experimental system; in the absence of this the authors should consider rephrasing their work to discuss "early gene markers of EndMT" or "EndMT early transcriptional program" and rephrase their abstract and introduction more carefully to indicate their focus on this aspect of the phenotype only.

We thank the reviewer for this suggestion. Accordingly, we properly labelled Snail1 and Serpine1 as early TF factors of EndMT and aSMA and Id1 as mesenchymal markers along all the manuscript. We rephrased abstract, introduction and discussion.

2) In multiple figures (1B, 1C, 1D, 2A, 2B, 2C, 3A, 4B, 5B, 5E, 6B, 6C, 6D, S3B) the authors appear to have used one-way ANOVA and ANOVA post-tests on normalized data. As ANOVA is an analysis of variance, including samples with no variance due to normalization artificially enhances statistical power and is inappropriate. The approach of figure S1B is better, where all the individual values are maintained and then normalized, allowing variance in the control group. I don't entirely agree with parametric testing (ANOVA rather than Kruskal-Wallis) in this setting either; however, this is commonly done, and I will defer it to the editor's/journal's discretion.

We thank the reviewer for this comment regarding the statistical analysis and we apologize for not having been enough clear in the explanation of our analysis pipeline. We agree with the objection that using normalized data can somehow enhance statistical power; therefore, all the experiments have been re-analyzed keeping the individual values from each replicate (i.e. Ct), and then normalized. Moreover, to meet the assumption of normal distribution required by parametric tests like ANOVA, all datasets have been first tested for normal distribution by applying the Shapiro-Wilk test; after that we decided to apply the parametric ANOVA (or Student's t-test) or the non-parametric variant Kruskal-Wallis (or Mann-Whitney) accordingly. For experiments where many genes were analyzed, we chose to apply the same test for all genes, in order to have more consistent results; in these cases, we applied the test that best fitted with the majority of the datasets. For instance, in Figure 1B, only 1 out of 24 datasets was not normally distributed ("KO-CTR" of Serpine1), therefore we applied the One-Way ANOVA for all. We relied on the fact that one-way ANOVA is considered a robust test against the normality assumption and it tolerates violations to this assumption rather well. On the other side, in Figure 6C we applied the Kruskal-Wallis test as 7 out of 8 datasets were non-normal distributed.
The statistical test applied for each plot is reported in the corresponding legend.

3) Similarly, the qPCR data that makes up much of the data in the paper should be presented and analyzed in log scale, as this is the scale on which the measurements (deltaCT/delta-deltaCT values) are performed. It is clear this has not been done based on the SEM error bars; curiously the manuscript does not at present contain a qPCR methods section so that I know what has been done in terms of analysis and seems to perform the statistics differently in different figures (as noted above). I am suspicious that considerable baseline downregulation of TGF-beta responsive genes in YAP KO cells and significant induction of those genes (from a low baseline) by TGF-beta is being obscured by the data as currently presented (in linear scale). For example, I suspect there is a statistically significant upregulation of Acta2,Fn1,and Serpine1 with TGF-beta induction (Figure 1a) when analyzed appropriately in log scale.

The 2^{-DCt} (or the similar 2^{-DDCt}) method has been the most widely used and accepted method to present RT-qPCR results and it was described in the Applied Biosystems User Bulletin No.2, released in 1997. In the last 2 decades, a bulk of literature was published supporting the use of

© 2021. Published by The Company of Biologists under the terms of the Creative Commons Attribution License (https://creativecommons.org/licenses/by/4.0/).

this method. Schmittgen and Livak in their work published in 2008 on Nature Protocols (and in another paper: Livak and Schmittgen, Methods 2001) showed how the use of this method is to be preferred when analyzing and presenting RT-qPCR results. They stated that: "*When real-time PCR data is to be presented as individual data points it should be presented as 2^{-DCt} or 2^{-Ct} rather than the raw Ct value. When presenting the data from gene expression profiling studies, one would always want to normalize the data to an internal control, so in this case the 2^{-DCT} is appropriate where DCt = (Ct gene of interest - Ct internal control)*". They also pointed the attention on the statistical analysis: "*It should be emphasized that statistical tests should not be run on the raw Ct data and s.d. should always be calculated after the 2^{-DDCt}, 2^{-DCt} or 2^{-Ct} transformation has been performed*".
Moreover, we use it to parallel the expression level of mRNA and the relative protein by western blot analysis, which must be analyzed as linear fold change. In this context, to be able to better compare results obtained by different techniques, the linear fold-change (2^{-DDCt}) gives the most consistent readout.
We agree with the reviewer that there is a partial baseline downregulation of TGF-ß responsive genes, however:
1) neither the baseline downregulation nor the slight increase upon stimulation are statistically significant with the One-Way ANOVA applied,
2) the expression level of these genes in KO cells upon TGF-ß stimulation is significantly lower than in WT cells. We therefore can conclude that in KO cells to TGF-ß triggers a lower transcription of target genes compared to WT cells.

4) The authors make a strong claim that their phenotype is YAP-specific, with no contribution from TAZ. However, they are using YAP KO cells, while their shRNA for TAZ demonstrates only about 60% reduction. I am quite concerned that Serpine1 and Acta2, genes that have known TEAD binding domains [Ota et al., Development 135, 4059-4069 (2008)] are not impacted by TAZ reduction at all, suggesting that either the knockdown is insufficient or TAZ is not driving TEAD transcriptional activation at all in these cells. What is the relative expression of Yap and TAZ in the cells (by CT value?). Is it possible that TAZ is just not very highly expressed? At a minimum, the authors' strong claims would be enhanced by demonstrating a TAZ-specific phenotype that is impacted (another canonical TEAD gene such as CTGF, CYR61, ANKRD1, etc. for example, or a decrease in a TEAD reporter assay). An even stronger approach would be to try overexpressing TAZ4SA in their knockout cells and show that this does not rescue their phenotype. Without additional data, I think they can only state that 2.5-fold reduction in TAZ levels did not have a strong impact on their phenotype in this cell line.

We agree with the reviewer that the downregulation of TAZ is not complete, as many times can happen with the shRNA-mediated approach, which is less efficient than a genetic KO. However, in our experiments, the shRNA showed a downregulation of TAZ around 75% both at the protein (note the new panel in Figure S2A) and mRNA levels (Figure S2B). The expression levels of YAP and TAZ in the cells are pretty comparable, with YAP being around 50% more expressed then TAZ, as assessed by both RT-qPCR (mean Ct YAP=24.26; TAZ 29.95; data not shown) and western blot (see Figure S2A). To confirm that the 75% reduction of TAZ we obtained was sufficient to affect its transcriptional co-regulator activity, we checked the expression level of a panel of YAP/TAZ target genes (*Cyr61*, *Ptgs2* and *Ankrd1*; Venkataramani V et al., 2017), which resulted to be reduced upon the deletion of YAP and the KD of TAZ in our cells. As shown in the new version of Figure S2B, the KD of TAZ induced a strong downregulation of Cyr61(-71% TAZ-KD; -90% YAP-KO) and Ptgs2 (-97% TAZ-KD; -95% YAP-KO) comparable to the KO of YAP, while Ankrd1 was less affected by TAZ-KD than by YAP-KO (-39% TAZ-KD; -98% YAP-KO).
Overall, we think that these new data support the concept that, in our condition, the TAZ-KD is sufficient to inhibit its biological activity.

Minor Comments:

1) Page 6 - I am not sure the relevance of the Qin paper to your manuscript other than being more recent - it mostly makes me wonder whether the authors should have looked at Smad7 themselves. It might be better for it to be included in the list of references and a more general discussion that YAP and TGFb/Smad3 interactions are well-established in other cell systems.

© 2021. Published by The Company of Biologists under the terms of the Creative Commons Attribution License (https://creativecommons.org/licenses/by/4.0/).

We thank the reviewer for this comment. We agree with the reviewer and we moved this reference in the list related to a more general discussion of Yap and Smad3. Following the suggestion, we also analyzed the level of expression of SMAD7 as now reported in Fig.1 panel D.

2) The current text doesn't seem to grapple much with the reduction in Smad3 mRNA (demonstrated in figure 2B and 6B) in the YAP KO line. The data showing that reintroduction or increase in nuclear YAP prevents degradation (via GSK) [fig 2C, 5A-C] is solid, but the authors do not address or speculate about why YAP KO cells have lower transcriptional SMAD3 activity, or whether this impacts interpretation of any of their data (in particular Figure 3A).

We agree with the reviewer, we did not comment on the lower SMAD3 mRNA level in YAP KO cells. Given the observation that in YAP KO cells *Smad3* mRNA levels were lower compared to the WT counterpart and that upon the introduction of YAP-5SA this level was not rescued in KO cells nor increased in WT (Figure 2B), we can speculate that YAP is involved in SMAD3 mRNA espression, but not in a direct way does. According with his/her suggestion, we now commented this data in the Results and Discussion sections.

3) Page 11 - The suggestion that YAP phosphorylation stabilization means there is no nuclear accumulation of YAP is not really accurate (it does, however, suggest no activation of canonical Hippo pathway signaling). There has been significant evidence that mechanical cues, for example, do not require changes in YAP phosphorylation to induce nuclear YAP shuttling (see Elosegui-Artola et al., Cell Volume 171, Issue 6, 30 November 2017, Pages 1397-1410.e14). The nuclear/cytoplasmic fractionation is much more convincing that there are not strong changes in YAP localization with TGF-beta stimulation. A small change of phrasing would be helpful.
We thank the reviewer for giving us the possibility to clarify this aspect. We checked by WB the phosphorylation of YAP on Ser127, since it is responsible for its cytoplasmic retention in both canonical and not-canonical Hippo signaling pathways (Basu, 2003; Giampietro, 2015; Moon, 2017; Zhao, 2007), but this analysis is only suggestive that TGFβ was not inducing Yap re-localization into the nucleus. To definitely prove this hypothesis, we performed nucleus/cytoplasmic fractionation and immunofluorescence analysis. According to the reviewer suggestion we rephrased the paragraph in the Results.

4) There are no immunofluorescence methods or reference to such methods. The YAP staining is fairly unconvincing - a positive control for nuclear ocalization (for example, the YAP5SA cells) would be helpful.

We apologize that the IF methods were missing. We now added them to the Materials and methods section. Moreover, we improved the quality of YAP staining and, for more clarity, we included both a negative and a positive control for the staining. As negative control of the specificity of the Ab, we stained YAP-KO cells; while as positive control of the nuclear staining of YAP we chose a more physiological condition: we stained WT cells cultured in sparse conditions, where YAP has been shown to accumulate into the nucleolus (Giampietro et al., JCB 2015). These new data have been included in the new version of Figure 4 panel C.

5) In figure 5E, why is Serpine1 not stimulated in the NaCl condition? Why was the immunofluorescence performed at 2h while the qPCR performed at 24hours? If it is known by western, N/C fractionation, or IF that the nuclear (or total) accumulation of SMAD3 persists between 2 and 24h of LiCl treatment in the KO cells it should be shown or at least stated.

To answer to the first question: there is actually an upregulation of both *Fn1* and *Serpine1* upon TGFβ stimulation in the NaCl condition; however, this upregulation is "masked" in the statical analysis, if we apply the ANOVA test, because of the very high expression level of *Fn1* and *Serpine1* in the LiCl condition. In fact, if we compare WT-NaCl with or without TGFβ (columns 1 and 2 of each plot reported below in Fig. Rebuttal 1) applying the non-paired, two-tailed Student's t-test, both *Fn1* and *Serpine1* show a statistically significant increase upon TGFβ stimulation. This confirms that the response of the cells in this experimental set-up is in line with what previously described (Figure 1 of the paper), although the complexity of the multiple comparisons can sometimes weaken the statistical significance. Moreover, if we compare the KO cells stimulated with TGFβ and treated with either NaCl or LiCl, by applying the non-paired, two-tailed Student's t-test, neither *Fn1* nor *Serpine1* shows statistically

© 2021. Published by The Company of Biologists under the terms of the Creative Commons Attribution License (https://creativecommons.org/licenses/by/4.0/).

significant increase upon TGFß stimulation, therefore confirming that the inhibition of GSK3ß up-regulates *Fn1* and *Serpine1* mRNA expression in WT but not in KO cells in response to TGFß.

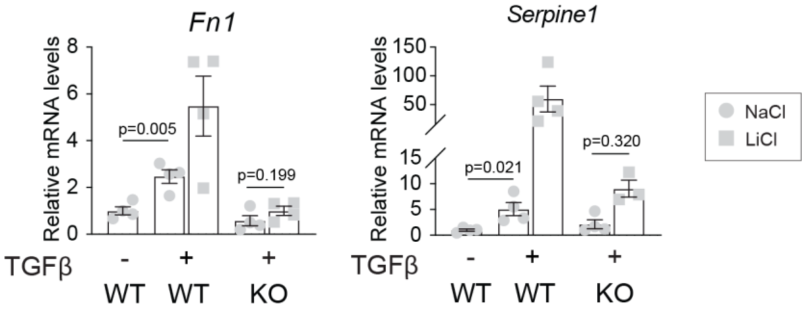

Fig Rebuttal 1: P-values obtained when performing statistical analysis applying the non-paired, two-tailed Student's t-test

To answer to the second question: we previously reported that in endothelial cells peak levels for SMAD3 phosphorylation were detected by WB between 45 and 80 min of stimulation with TGF-ß1 (Rudini et al., 2008; Maddaluno et al., 2013), nuclear SMAD3 accumulation by IF after 2 hours (Rudini et al., 2008), and SMAD-dependent transcription by RT-qPCR after 24 hours (Maddaluno et al., 2013). Moreover, in endothelial cells, LiCl treatments have been proved to affect gene transcription after an O/N incubation (Taddei et al., 2008). This are the reasons why these specific time points were selected in our experimental set up.

6) Figure 6A should include either the IgG controls for each set of cells or the Y-axis needs to labeled properly to explain the normalization performed based on the methods. It would be preferable to show the IgG controls as in Zhang et al (2014). Experimental triplicates should not have error bars; if 3 independent experiments were done, then the data should be pooled and shown.

We thank the reviewer for pointing out this issue. In the old graph only one representative ChIP qPCR assay (out of 3 performed) was reported. The ChIP IgG values were subtracted by the values of YAP and SMAD3 ChIP respectively, and the error bars reported were from the technical triplicates of the qPCR assay.
Following the suggestion of the reviewer, in the new Figure 6 panel A we pooled together the data from the 3 biological independent experiments performed, we showed the IgG values and we properly labelled the Y-axis.

7) There is some confusing language in the text surrounding YAP as a "co-transcriptional factor" for SMAD3 and "binding" to the Smad3 promoter. YAP is a transcriptional co-regulator (transcriptional cofactor is acceptable language as well), and does not bind DNA directly. The authors have shown YAP-SMAD3 binding directly and indirectly throughout this paper, and their data suggests it allows for increased nuclear accumulation and stabilization of SMAD3. Their CHIP assay demonstrates YAP and Smad3 are both present at the Fn1 promoter site. It would be helpful to adjust the text for clarity.

Following the reviewer's suggestion, we have corrected "co-transcriptional factor" with "transcriptional co-regulator" throughout all the text.

8) The authors at times appear to overstate the requirement for both YAP and SMAD3 for transcriptional induction of their genes of interest. The data show fair induction of Fn1 and Serpine1 in the SMAD3+/YAP-/TGFb, YAP+/siSMAD3/TGFb, and unstimulated YAP5SA conditions in figure 6, suggesting a co-operative, additive, or possibly synergistic effect on the TGF-beta mediated transcription of these genes (neither is 100% essential). I would recommend looking over the text generally to make sure that statements about things that are "essential" or "required" are fully backed up by the data.

We agree with the interpretation of a co-operative role of YAP and SMAD3 rather than one of them being "essential" to drive early EndMT program. According to the suggestion, we edited the text through all the manuscript.

© 2021. Published by The Company of Biologists under the terms of the Creative Commons Attribution License (https://creativecommons.org/licenses/by/4.0/).

9) The authors have not ruled out the possibility that YAP is directing TEAD-mediated transcription of the TGF-beta responsive genes as an important factor, rather than enhancing SMAD3 transcription directly (as opposed to indirectly through nuclear sequestration or stabilization). Certainly Hiemer et al. would suggest TEAD binding is important for Serpine1 transcription, for example. A brief discussion of this point (or an experiment to rule out TEAD-mediated effects, such as using YAP5SA-S94A expressing cells) is required.

We thank the reviewer for this input. According to his/her suggestion, to rule out the role of TEAD in the molecular mechanisms we identified, we silenced TEAD1 in our cells and we verified by RT-qPCR that the downregulation we obtained was able to significantly reduce the expression level of TEAD target genes. We then evaluated the expression level of *FN1, Serpine1* and *Snai1* and we did not get any significant inhibition upon TGFß stimulation. This suggests that YAP and SMAD3 exert a cooperative effect independently on TEAD. This set of data is now reported in the new Figure S3 and commented in the Results section.

10) The authors should comment on the fact that using an immortalized cell line to assay pro-proliferative phenotypes (EndMT) involving a known oncogene (YAP) has some inherent limitations.

We have already used in the past polyoma Middle T to generate many immortalized endothelial cell lines that have been extensively characterized in previous papers from our lab (Maddaluno et al 2013; Bravi et al 2015; Cuttano et al. 2016; Giannotta et al., 2014; Giampietro et al., 2015; Giampietro, Deflorian et al., 2015).
We agree with the reviewer that the use of immortalized cell line have some limitations. In the revised version of the paper, we added a better characterization of the endothelial and phenotypic characteristics of the 2 cell lines. First, we showed that both cell lines retained their ability to form mature monolayers, shown by the presence of both adherens and tight junction markers, as well as their endothelial identity. Moreover, we verified that the established *in vitro* systems retained the major characteristics of the *in vivo* models (in terms of proliferation and migration) (New Figure S1). Finally, we performed TGFß treatment on primary ECs freshly-isolated from WT adult mice in which YAP was knock-down by shRNA. As shown in new Figure S4, using lentiviral vector-delivered shRNA we could downregulate YAP expression of around 60% which resulted in a 30% inhibition of *Serpine1* activation upon 24h treatment with TGFß. This data showed a direct correlation between the expression level of YAP and the response to TGFß also in primary endothelial cells, thus supporting the fact that that the immortalized cell lines can be used taking into consideration eventual tradeoffs. This statement has been added and commented in the manuscript in the Results section.

Reviewer 2 Advance Summary and Potential Significance to Field: The manuscript is a detailed biochemical study of the role of YAP in SMAD3-dependent signaling and EndoMT. The findings are novel and relevant to a field which has been growing over the last few years because of the potential implications of EndoMT in many human pathologies.

Reviewer 2 Comments for the Author:

This is an overall well conducted study, with some limitations discussed below.

We thank the reviewer for the careful reading of our manuscript.

1. Immortalized mouse lung EC are used in this study. The Authors should provide basic EC characterization with images of the cells by IF and PRC to show how much of the endothelial phenotypes is retained. This is important in general, but more so here given that the claim to novelty is the effect on the endothelial lineage; the pathways under investigation have already been described other lineages.

We agree with the reviewer that the use of immortalized cell line have some limitations. In the revised version of the paper, we added a better characterization of the endothelial and phenotypic characteristics of the 2 cell lines. First, we showed that both cell lines retained their ability to form mature monolayers, shown by the presence of both adherens and tight

© 2021. Published by The Company of Biologists under the terms of the Creative Commons Attribution License (https://creativecommons.org/licenses/by/4.0/).

junction markers, as well as their endothelial identity. Moreover, we verified that the established *in vitro* systems retained the major characteristics of the *in vivo* models (in terms of proliferation and migration) (New Figure S1).

2. To exclude possible bias from immortalised EC lines, another endothelial model of YAP ko, perhaps by siRNA, should be used to validate some of the key findings in HUVEC or another human primary endothelial cell.

We have already used in the past polyoma Middle T to generate many immortalized endothelial cell lines that have been extensively characterized in previous papers from our lab (Maddaluno et al 2013; Bravi et al 2015; Cuttano et al. 2016; Giannotta et al., 2014; Giampietro et al., 2015; Giampietro, Deflorian et al., 2015).

Following the suggestion of the reviewer, we have performed the key experiment of TGFß stimulation after YAP KD in murine primary endothelial cells. As shown in Figure S4, using lentiviral vector-delivered shRNA we could downregulate YAP expression of around 60% which resulted in a 30% inhibition of Serpine1 activation upon 24h treatment with TGFß. This data showed a direct correlation between the expression level of YAP and the response to TGFß. Nevertheless, to address reviewer's request, a similar experiment was performed using human primary endothelial cells such as HUVEC and Human Pulmonary Microcirculation EC (HPMEC). Unexpectedly, we encountered several technical limitations:
1) TGFß is contained in the serum; therefore, TGFß stimulation must be performed under serum starvation condition in order to induce a response in the cells;

2) primary cells, especially HUVECs, are particularly sensitive to serum deprivation and the monolayer starts to be disrupted after 30-32 hours of starvation;

3) The integrity of a stabilized monolayer is critical for the localization of YAP (Giampietro et al., 2015) which in turn regulates the response to TGFß, as well as for the response of ECs to TGFß (Rudini et al., 2008).

In these experimental conditions, human primary endothelial cells did not respond properly to TGFß stimulation. Thus, we concluded that HUVEC and HPMEC are not a suitable model to analyze the response mediated by YAP upon TGFß treatment.

3. Fig1D: expression levels of Id1 and Serpine1, are used as readout of ALK1/SMAD1 and ALK5/SMAD3 signalling, respectively. Based on the regulation of these, they conclude "….. that TGFß downstream signaling is impaired in absence of YAP and that YAP is specifically required for SMAD3 but not SMAD1 signaling in EC." A few more targets of these pathways should be measured to support this statement.

***Id1* and *Serpine1* genes are widely considered specific target genes of the ALK1/SMAD1 and ALK5/SMAD3 signaling cascades respectively (Schmierer and Hill, Rev Mol Cell Biol 2007); in fact, previous papers have shown that:**

1) SMAD3 binds directly the promoter of *Serpine1* and induces its transcription (Dennler et al., The EMBO Journal 1998; Zawel et al., Molecular Cell 1998);
2) SMAD1 binds directly the promoter of *Id1* and induces its transcription (Korchynskyi and Peter ten Dijke, The Journal of Biological Chemistry 2002).
To meet the valuable suggestion of the reviewer, we tested other known targets of SMAD1 (*Id3)* and SMAD3 (*Smad7*), upon knock down of SMAD3 and TGFß stimulation. As shown in the new Figure 1D, only *Serpine1* and *Smad7* levels were regulated in a SMAD3-dependent manner. These results are discussed at in the Results section.

4. Page9 and Fig 2A: TGFb induced P of SMAD1 and 3, and YAP regulation of SMADs expression. Statistical analysis should be carried out between WT and KO, because it looks like the KO cells have nearly double the level of P SMAD3 in response to TGFb. The Authors should comment on this finding: why is Yap KO increasing TGFb-dependent SMAD1 and 3 Phosphorylation.

© 2021. Published by The Company of Biologists under the terms of the Creative Commons Attribution License (https://creativecommons.org/licenses/by/4.0/).

Given the new results above (Figure 1D), which added more evidence on the fact that YAP impacts only on SMAD3 and not SMAD1 TGFß-mediated gene transcription, we removed further data on SMAD1, whose role is beyond the scope of this paper.
We agree with the reviewer that there is indeed an increase in pSMAD3 in response to TGFß in KO vs WT cells, as confirmed by the statistical analysis, that we now included in the plots (Figure 2A). This increase paralleled the slight increase of the two TGFß receptors shown in Figure 1F. Therefore, these data support the idea that YAP is not required for the first events of the signaling cascade, i.e. the expression of receptors and/or the phosphorylation of SMADs, but it is important for the downstream transcriptional activity. We edited the text to better clarify this point. We can speculate that the slight increase of Tgfbr1 and Acvrl1 receptors and of pSMAD3 is a compensatory response of the cell to the impaired TGFß signaling pathway.

Also, the text on page 9 only focuses on regulation of SMAD3, but very similar effects are seen with SMAD1. This is in contrast with what stated above (Fig 1D). Does TNFb significantly increase P of SMAD1 but not SMAD3? Stats are missing. For the rest of the study, SMAD1 is ignored; but based on this initial finding, it seems that SMAD1 may be playing a role too. This needs more detailed investigation.

Upon stimulation with TGFß, the expression of SMAD1 target genes *Id1 and Id3* is increased at the same level in YAP KO and WT cells (Fig.1B, E). On the other hand, although SMAD3 is phosphorylated in response to TGFß (Fig.2A), the expression of its target genes *Serpine1* and *smad7* is inhibited in KO cells (Fig.1B, E). This suggests that the loss of YAP impaired the transcription activity mediated by the pathway TGFß/ALK5/SMAD3 but not by TGFß/ALK1/SMAD1. Therefore, we focused our investigation on the role of YAP in the SMAD3-mediated signaling pathway.
However, although not evident from our data, we cannot exclude that YAP could be a regulator of SMAD1 activity too, but this is beyond the scope of this paper.

5. Fig 2D Page 10: The Authors state "SMAD3 phosphorylation occurred at comparable levels between the two cell lines, suggesting that YAP did not influence SMAD3 C-term phosphorylation". But the figure shows statistical significance between WT and KO - that's confusing.

We apologize with the reviewer, the text was confusing. The statistical analysis shows indeed a statistical difference of both WT and KO cells at any time after stimulation if compared to the non-stimulated condition T0. On the other hand, there is no significant difference between WT and KO cells at any time point. We have better explained this concept in the figure legend and in the text.

6. An important aspect of this story seems to be the regulation of SMAD3 expression by YAP. Can the Authors speculate about the mechanism and the implications on their findings?

We agree with the reviewer, we did not comment on the lower SMAD3 mRNA level in YAP KO cells. Given the observation that in YAP KO cells *SMAD3* mRNA levels were lower compared to the WT counterpart and that upon the introduction of YAP-5SA this level was not rescued in KO cells nor increased in WT (Figure 2B), we can speculate that YAP is involved in SMAD3 mRNA expression, but not in a direct way. According with his/her suggestion, we now commented this data in the Results and Discussion sections.

Other comments:

1. R-SMADs are introduced on page 8 without any explanation of the abbreviation.

We apologies for this, we have now explained the abbreviation in the text.

2. General comment: in the Results, there is lengthy background information with references. The Result section should be revised and perhaps figures reorganised to allow for a more linear narrative. Some of the discussion on the relevant literature should be moved to introduction or discussion (example: the first 8 lines of the results)

© 2021. Published by The Company of Biologists under the terms of the Creative Commons Attribution License (https://creativecommons.org/licenses/by/4.0/).

Following the reviewer suggestion, the background information with references have been removed from the results, and used in the discussion and introduction.
3. All bar graphs: The symbols are very small and very similar to each other, making it difficult to discriminate between them. Please use larger symbols, and more clearly different between each other.

Following the reviewer suggestion, we have increased the size of symbols in every plot to make them more visible.

4. Figures are not described with enough detail. Example: Fig 6B. The figure has 3 different graphs, each with 8 conditions, with very small legends. The reader has to struggle through it all without much explanation. The Authors should check all their figure description in the text and improve where necessary.

Following the reviewer suggestion, we modified the figure legends improving the text.

5. Fig S1 and S2 shows that SMAD3 binds YAP. This is new data important for this story, and should be in the main paper.

Following the reviewer suggestion, we moved to the main Figure 4 the Co-IP showing the physical binding between YAP and SMAD3.

6. Fig 4E: TGFb treatment and YAP/SMAD3, showing a representative image of 3. Quantification of all experiments should be shown, given that the effect seems pretty small.

We agree with the reviewer that the differences are pretty small. We also think that while western blot is at best semi-quantitative, to interpret bands on a blot by densitometry as quantitative expression values is inaccurate when you're doing an IP beforehand. For this reason, we removed from the manuscript any comment and speculation on the differences obtained by the TGFß treatments, since they are not relevant for the message of the paper.

7. Fig 5A shows that SMAD3 Phosphorylation upon TGFb time course is higher in YAP ko cells. They already showed that in Fig 2A, without the timecourse: the data should be reorganised for clarity and consistency

We thank the reviewer for this comment that show that our description of the results was not clear enough. Indeed, Figure 2A and Figure 5A show the phosphorylation of SMAD3 in two different residues which mediate two different outcomes of the SMAD protein. Figure 2A shows the phosphorylation of serine residues 423/425 at the C-term region, which triggers the translocation of SMAD3 from the cytoplasm to the nucleus. Figure 5A, instead, shows the phosphorylation of serine residue 204 that triggers proteasomal degradation. These two figure panels give different biological messages and this is the reason why they are in different figures. While in Figure 5A pSMAD3 S204 was clearly indicated, Figure 2A, 2D as well as Figure 4 were missing the phosphorylated residues, thus triggering this misunderstanding. We corrected all the figures adding the label pSMAD3 S423-425 where missing.

8. Fig 6B shows that ko of SMAD3 reduces levels of the two target genes analysed in WT but not YAP ko cells. The graph shows great variability in the Fn1 samples for the KO + siControls. The conclusions would be more reliable with a few more datapoints. On this figure, the text states: "Data showed that SMAD3 is necessary for inducing both Fn1 and Serpine1 expression upon TGFß stimulation, further indicating that SMAD3 is important for driving EndMT gene transcription." But we knew this already; this is not the conclusion from this figure, which aims to investigate the possible synergy between SMAD3 and YAP.

We have repeated the experiment as suggested to increase the sample size. The statistical analysis revealed that the deletion of YAP or SMAD3 reduces the levels of *Fn1* and *Serpine1* after TGF□ treatment with the same efficacy: i.e. WT-siSMAD3 vs WT-siSCR p<0.01; KO-siSCR vs WT-siSCR p<0.01; WT-siSMAD3 vs KO-siSCR p>0.05. On the other hand, the double deletion of YAP and SMAD3 induced a further reduction of *Fn1* and *Serpine1*, although slight, thus

© 2021. Published by The Company of Biologists under the terms of the Creative Commons Attribution License (https://creativecommons.org/licenses/by/4.0/).

suggesting a possible collaborative or synergistic role of YAP and SMAD3 that has been commented in the Results and discussion sections.

9. The shortcut "ChIP assay" is used here for ChIP qPCR, which is the correct term.

We have corrected the term throughout all the text.

Reviewer 3 Advance Summary and Potential Significance to Field:

The manuscript by Savorani et al., focuses on the role of the co-transcriptional regulator YAP in TGFbeta-induced endothelial to mesenchymal transition (EndMT). Given the role of YAP signaling in orchestrating TGFbeta signaling in multiple cell types, the authors assessed weather loss of YAP in endothelial cells (ECs) blocked EndMT induced by TGFbeta. Using an immortalized endothelial cell line derived from mouse lungs the authors employed a series of biochemical approaches and found that YAP plays a role in SMAD3 protein stability and consequently TGFbeta-induced EndMT. YAP prevented GSK3beta-promoted SMAD3 phosphorylation and degradation. Loss of YAP favored SMAD3 degradation and inhibited TGF-beta signaling. Overall the study is well presented and results are adequately discussed. The novelty of the study, however, is compromised by the existing literature reporting YAP as a SMADs cofactor that plays a role in TGF-beta signaling in multiple cell types.

We thank the reviewer for the careful reading of our manuscript.

Reviewer 3 Comments for the Author:
Given the emerging role for YAP in regulating sprouting angiogenesis, the authors should elaborate on how their findings reconciliate with YAP angiogenic functions. For instance, does VEGF signaling interfere with YAP-promoted EndMT in presence of TGFß? Can the author speculate on how YAP controls endothelial migration in the contest of EndMT and angiogenesis?

We thank the reviewer for this comment. We agree that, besides the already mentioned pathophysiological relevance of the mechanism described in this paper, these findings are important because of their possible connection to other receptor-mediated pathways. Indeed, it has been proved that YAP is a central mediator of VEGF signaling in endothelial cells where it contributes to angiogenesis (Wang et al., 2017; Azad et al., 2018; Elaimy et al., 2018) as well as in tumor cells (Zanconato et al., 2016; Elaimy et al., 2018). We added comments on this aspect in the discussion, speculating that since both VEGF and TGFß signaling pathways are tightly regulated during development and aberrantly activated in pathologies, the impact of this work is likely to contribute the interest in targeting YAP as a therapeutic approach to inhibit converging molecular pathways.

EndMT is associated with the downregulation of junctional proteins, including VE-cadherin, to facilitate endothelial dissociation and mesenchymal differentiation. Does YAP compromise vascular integrity and cellular junctions? The authors should also show endothelial marker staining to ensure cell maintained their endothelial identify following immortalization. Junctional protein staining would also help to validate endothelial integrity before treatments.

We have already used in the past polyoma Middle T to generate many immortalized endothelial cell lines that have been extensively characterized in previous papers from our lab (Maddaluno et al 2013; Bravi et al 2015; Cuttano et al. 2016; Giannotta et al., 2014; Giampietro et al., 2015; Giampietro, Deflorian et al., 2015). Following immortalization, the cells maintain their endothelial identity in terms of morphology, monolayer formation and gene expression. We have added in the revised version of the manuscript the characterization of the endothelial phenotype on our immortalized cell lines. As shown in the new Figure S1, immortalized WT and KO endothelial cells form a continuous monolayer and express the markers of adherens and tight junctions. Moreover, they collectively migrate to close the gap when challenged in a classical scratch assay. YAP-KO cells showed a reduction in Claudin5 expression, as previously observed *in vitro* and *in vivo* (Choi et al., 2015; Kim et al., 2017). Moreover, the loss of YAP impairs both the migration and proliferation speed as previously reported (Choi et al., 2015; Kim et al., 2017). All these data confirm that our cell lines maintained their endothelial

© 2021. Published by The Company of Biologists under the terms of the Creative Commons Attribution License (https://creativecommons.org/licenses/by/4.0/).

identity following immortalization. These new data have been included in the new Figure S1 and commented in the Results section.

My major concern in this study is that the authors do not use primary endothelial cells to demonstrate or corroborate their major findings. The use of an immortalized cell line to evaluate EndMT should be accompanied by further experiments using primary endothelial cells as a model. Given the contribution of EndMT is multiple human disorders, the use of human endothelial cells would strengthen the findings of the study and provide insights on potential therapeutic implications for human disease. Key findings of the study should be reproduced in primary cells.

Following the suggestion of the reviewer, we have performed the key experiment of TGFß stimulation after YAP KD in murine primary endothelial cells. As shown in Figure S4, using lentiviral vector-delivered shRNA we could downregulate YAP expression of around 60% which resulted in a 30% inhibition of Serpine1 activation upon 24h treatment with TGFß. This data showed a direct correlation between the expression level of YAP and the response to TGFß.
We also performed the same experiment on two human primary endothelial cells i.e. HUVEC and HPMEC (Human Pulmonary Microcirculation EC); however we encountered several technical limitations:
1) TGFß is contained in the serum; therefore, TGFß stimulation must be performed under serum starvation condition in order to induce a response in the cells;
2) primary cells, expecially HUVECs, are particularly sensitive to serum deprivation and the monolayer starts to be disrupted after 30-32 hours of starvation;
3) the integrity of a stabilized monolayer is fundamental for the cells to respond to several stimuli, including TGFß (Rudini et al., 2006).
For all these reasons, we couldn't manage to obtain a good stimulation with TGFß together with the preservation of the monolayer integrity. Thus, we concluded that HUVEC and HPMEC are not a suitable model to analyze the response mediated by YAP upon TGFß treatment.

Second decision letter

MS ID#: JOCES/2020/251371

MS TITLE: A Dual Role of YAP in Driving TGFβ[beta]-mediated EndMT

AUTHORS: Cecilia Savorani, Matteo Malinverno, Roberta Seccia, Claudio Maderna, Monica Giannotta, Linda Terreran, Eleonora Mastrapasqua, Stefano Campaner, Elisabetta Dejana, and Costanza Giampietro

ARTICLE TYPE: Research Article

We have now reached a decision on the above manuscript.

To see the reviewers' reports and a copy of this decision letter, please go to: https://submit-jcs.biologists.org and click on the 'Manuscripts with Decisions' queue in the Author Area. (Corresponding author only has access to reviews.)

As you will see, the reviewers feel that overall you did a good job addressing their earlier comments, but raised some critical points that will require amendments to your manuscript. I hope that you will be able to carry these out because I would like to be able to accept your paper, depending on further comments from reviewers.

We are aware that you may be experiencing disruption to the normal running of your lab that makes experimental revisions challenging. If it would be helpful, we encourage you to contact us to discuss your revision in greater detail. Please send us a point-by-point response indicating where you are able to address concerns raised (either experimentally or by changes to the text) and where you will not be able to do so within the normal timeframe of a revision. We will then

© 2021. Published by The Company of Biologists under the terms of the Creative Commons Attribution License (https://creativecommons.org/licenses/by/4.0/).

provide further guidance. Please also note that we are happy to extend revision timeframes as necessary.

Please ensure that you clearly highlight all changes made in the revised manuscript. Please avoid using 'Tracked changes' in Word files as these are lost in PDF conversion.

I should be grateful if you would also provide a point-by-point response detailing how you have dealt with the points raised by the reviewers in the 'Response to Reviewers' box. Please attend to all of the reviewers' comments. If you do not agree with any of their criticisms or suggestions please explain clearly why this is so.

Reviewer 1

Advance summary and potential significance to field

In this work, Savorani, Malinverno, and their co-authors demonstrate YAP-Smad3 complex formation and cross-talk yielding synergistic induction of TGF-beta responsive genes in immortalized lung-derived endothelial cells. YAP has been shown to be of importance to EndMT in vivo and in vitro previously (Zhang et al.) via YAP-SMAD3 transcriptional interactions. The Varelas lab and others have also demonstrated the existence of TGF-beta - YAP/TAZ crosstalk via Smad3 interactions in epithelial and stem cell systems. These authors validate YAP- Smad3 complex formation and transcriptional synergy, but also demonstrate that YAP-SMAD3 binding prevents GSK-mediated SMAD3 phosphorylation and degradation to enhance SMAD3 nuclear localization and possibly contribute to gene transcription. The authors suggest Hippo/YAP pathway targeting may offer an alternative approach to targeting EndMT (and presumably other TGF-beta dependent processes) in endothelial cells.

Comments for the author

I would like to thank the authors for the time and care they took in an attempt to address my prior concerns. I think the work in its current form has some limitations that require some softening of wording and a limitations paragraph in the discussion, but do not anticipate additional experimental work.

Major Comments:

1) The role of TAZ. Based on the author's response, the cells express ~ 50 times the level of YAP than TAZ (CT value 24.26 vs. 29.95). It is somewhat impressive that TAZ knockdown does impact the expression of some TEAD-dependent genes in this setting - this may be due to relatively little nuclear signal in unstimulated confluent cells. Absent experiments involving overexpression of TAZ and/or CO:IP with TAZ, it is not possible to conclude that TAZ could not synergize with TGF-beta similar to YAP if it were more highly expressed. I would adjust the text to reflect that TAZ does not appear to be playing a role in mediating synergy with TGF-beta signaling in these cells, but that lower levels of expression could be playing a role in this phenomenon.

2) The role of TEADs. There are 4 TEADs expressed in mammalian cells (TEAD1-TEAD4). Is TEAD1 much more highly expressed in these cells than the others? It is interesting that the TGF-beta-mediated upregulation of some TEAD-regulated genes (Cyr61, PTGS2, ANKRD1) was very TEAD1 dependent; however, the lack of large changes in response to 50% knockdown of TEAD1 is a pretty modest argument against a TEAD-dependent effect more generally. Serpine1 has a known TEAD4 binding site (e.g. Marquard et al., cell communications and signaling, 2020).

Hiemer et al have likewise specifically shown TEAD2 and TEAD4 bind to Smad3.

Based on Figure S3, there does in fact appear to be some synergy between YAP and TEAD1 knockdown even in the data presented for the SMAD3-dependent genes and the SMAD3-regulated genes (expectedly) appear to be less dependent on either YAP or TEAD for their TGF-beta-associated upregulation than the canonical TEAD-dependent transcripts. I am not sure why TEAD involvement in the transcriptional regulation (as part of the nuclear complex) would necessarily be problematic in the authors' model; this mechanism is already suggested by the data from Hiemer et

© 2021. Published by The Company of Biologists under the terms of the Creative Commons Attribution License (https://creativecommons.org/licenses/by/4.0/).

al. On some level, I think it is a question that could be experimentally assessed via multiple TEAD knockdown or use of YAP5SA-S94A expressing cells. However, I think it would be better at this point to include in the discussion that TEADs may be part of the YAP-Smad3 nuclear complex and/or that TEAD-mediated transcription could be contributing to the synergistic induction of SMAD3-associated genes. I don't find figure S3 particularly compelling.

3) Statistical analysis. I greatly appreciate your change to using individual values in the statistical analysis; however, I remain mildly confused by the statistical approaches taken in the paper. Most of the analyses would seem to be appropriate for two-way ANOVAs looking at genotype (YAP KO vs. Control) and/or genotype x treatment interactions (KO vs. control x TGF-b), rather than a simple one-way ANOVA. I guess the current approach is not incorrect, but the ANOVA result is not very interesting and the readers need to look at the post-tests for useful information. The use of Fisher's LSD tests for post-tests is OK for exploratory data when labeled clearly as the authors have done; it is of course less rigorous than any correction for multiple testing. I think the argument regarding using log scale vs. fold expression is not settled in the literature; while CT values would not be expected to be normally distributed they are the directly measured variable. Other authors (such as Yuan et al., BMC Bioinformatics 2006 PMID 16504059) suggest performing the statistical analysis using the measured variable. Given that this appears to be an area of continued controversy, I withdraw my objection. The authors should include their qPCR analysis description as part of their methods (at present it must be extrapolated from CHIP-qPCR). Finally, it is appreciated that data that failed normalization tests were analyzed with non-parametric methods; lack of failure is not a strong guarantee of normality with such small numbers, but (in my opinion) attempted reanalysis at this point would require additional replicates and be prohibitively time-intensive.

Minor comments:

Figure S1F: I do not understand the notation on the 2-way ANOVA results.
Presumably the two-way is for time and genotype. The ANOVA should be statistically significant for time, possibly genotype and definitely time x genotype interaction, and I would report all 3 values. It would seem the relevant post-hoc comparisons would be between the 2 groups at the same timepoint, not to t=0.

Figure S4: I do not understand how n=3 experiments has produced n=8 datapoints. I think this is fairly weak evidence for an effect in primary cells. A sentence or two on the limitations of immortalized lines and why they were advantageous for use in this study (along the lines that have been argued in the rebuttal) would strengthen the paper.

Page 20: The discussion text states that "LiCl treatment was capable of increasing total SMAD3 protein level accumulation along with EndMT gene expression in TGFB-stimulated YAP KO cells." LiCl wasn't able to increase EndMT gene expression in KO cells - in fact, it is interesting that YAP appears to increase Smad3 levels and nuclear localization in a pS204 / GSK3B-dependent manner, but that reversal of this is insufficient to allow transcription of these TGF-beta-dependent / EndMT genes, indicating the need for synergistic transcriptional activity. This has been commented on earlier in the discussion (page 19); however I would suggest changing this part of the discussion to follow the paper - first findings about SMAD3 protein levels, next findings about sMAD3 turnover, and last findings about transcriptional synergy. This would require moving the end sentences of the large paragraph on page 19 to page 20.

Reviewer 3

Advance summary and potential significance to field

The authors have addressed my concerns except the validation of their major findings in primary human endothelial cells. See my comments below:

Comments for the author

The author claim that because of the presence of TGFb in the serum of the culture media, serum starvation is required in order to achieve an appreciable TGFb stimulation. While TGFb is present in serum (very low concentration), it is usually in its latent form or is bound to blood proteins such as

© 2021. Published by The Company of Biologists under the terms of the Creative Commons Attribution License (https://creativecommons.org/licenses/by/4.0/).

macroglobulin and then is inactive. Also, the majority of commercially available human endothelial cell culture media contain 5% FBS or less, further reducing the presence of active TGFb. Because the concentration of TGFb in serum is significantly lower than the recombinant one (5ng/ml) that was used to treat cells, the exogenous TGFb should activate this signaling even in presence of 5% serum. Alternatively, to reduce any potential contamination from active TGFb the author should reduce the serum concentration to 0.5-1% which should maintain endothelial monolayer for at least 18-24 hours (which is a sufficient time for TGFb stimulation). In addition there are numerous papers in which EndoMT was obtained in vitro in TGFb-treated HUVECs and other primary human endothelial cells. Here is one example (PMID: 17194892).

Second revision

Author response to reviewers' comments

RESPONSE TO REVIEWERS

Reviewer 1 Advance Summary and Potential Significance to Field:

In this work, Savorani, Malinverno, and their co-authors demonstrate YAP-Smad3 complex formation and cross- talk yielding synergistic induction of TGF-beta responsive genes in immortalized lung-derived endothelial cells. YAP has been shown to be of importance to EndMT in vivo and in vitro previously (Zhang et al.) via YAP-SMAD3 transcriptional interactions. The Varelas lab and others have also demonstrated the existence of TGF-beta- YAP/TAZ crosstalk via Smad3 interactions in epithelial and stem cell systems. These authors validate YAP- Smad3 complex formation and transcriptional synergy, but also demonstrate that YAP-SMAD3 binding prevents GSK- mediated SMAD3 phosphorylation and degradation to enhance SMAD3 nuclear localization and possibly contribute to gene transcription. The authors suggest Hippo/YAP pathway targeting may offer an alternative approach to targeting EndMT (and presumably other TGF-beta dependent processes) in endothelial cells.

Reviewer 1
Comments for the Author: I would like to thank the authors for the time and care they took in an attempt to address my prior concerns. I think the work in its current form has some limitations that require some softening of wording and a limitations paragraph in the discussion, but do not anticipate additional experimental work.
We thank the reviewer for the positive comments and appreciation for the work performed during the first round of revision and for the recognition that the work does not need additional experimental work.

Major Comments:
1) The role of TAZ. Based on the author's response, the cells express ~ 50 times the level of YAP than TAZ (CT value 24.26 vs. 29.95). It is somewhat impressive that TAZ knockdown does impact the expression of some TEAD- dependent genes in this setting - this may be due to relatively little nuclear signal in unstimulated confluent cells. Absent experiments involving overexpression of TAZ and/or CO:IP with TAZ, it is not possible to conclude that TAZ could not synergize with TGF-beta similar to YAP if it were more highly expressed. I would adjust the text to reflect that TAZ does not appear to be playing a role in mediating synergy with TGF-beta signaling in these cells, but that lower levels of expression could be playing a role in this phenomenon.
We agree with the reviewer that in these cell system, in confluent and unstimulated control condition, the low TAZ nuclear signal might impact on the expression of the TEAD-dependent genes. We also agree, that it cannot be excluded that higher levels of TAZ expression could synergize with TGF-beta similar to YAP. According with the reviewer suggestion we highlighted this aspect in the Results section at pag 8.

2) The role of TEADs. There are 4 TEADs expressed in mammalian cells (TEAD1-TEAD4). Is TEAD1 much more highly expressed in these cells than the others? It is interesting that the TGF-beta-

© 2021. Published by The Company of Biologists under the terms of the Creative Commons Attribution License (https://creativecommons.org/licenses/by/4.0/).

mediated upregulation of some TEAD-regulated genes (Cyr61, PTGS2, ANKRD1) was very TEAD1 dependent; however, the lack of large changes in response to 50% knockdown of TEAD1 is a pretty modest argument against a TEAD-dependent effect more generally. Serpine1 has a known TEAD4 binding site (e.g. Marquard et al., cell communications and signaling, 2020). Hiemer et al have likewise specifically shown TEAD2 and TEAD4 bind to Smad3. Based on Figure S3, there does in fact appear to be some synergy between YAP and TEAD1 knockdown even in the data presented for the SMAD3-dependent genes, and the SMAD3-regulated genes (expectedly) appear to be less dependent on either YAP or TEAD for their TGF-beta-associated upregulation than the canonical TEAD-dependent transcripts. I am not sure why TEAD involvement in the transcriptional regulation (as part of the nuclear complex) would necessarily be problematic in the authors' model; this mechanism is already suggested by the data from Hiemer et al. On some level, I think it is a question that could be experimentally assessed via multiple TEAD knockdown or use of YAP5SA-S94A expressing cells. However, I think it would be better at this point to include in the discussion that TEADs may be part of the YAP-Smad3 nuclear complex and/or that TEAD-mediated transcription could be contributing to the synergistic induction of SMAD3-associated genes. I don't find figure S3 particularly compelling.
We welcome the suggestion of the reviewer. Indeed, functions of YAP have been mainly attributed to its interaction with TEAD family of transcription factors (Walko, G. et al.,2017; Zhao, B. et al., 2008; Zhang, H. et al., 2009; Liu-Chittenden, Y. et al., 2012). Since TEAD1 is a primary mediator of YAP1-dependent gene regulation (Ota and Sasaki, 2008; Stein et al., 2015) and it has been demonstrated that a YAP-TEAD1 signaling controls angiogenesis (Mammoto et al., 2018), we looked for a role of TEAD1 and we found that it was not involved in this mechanism, although a more general TEAD-dependent effect cannot be excluded. For this reason, we added the latter comment in the Discussion section at pag 17-18.

3) Statistical analysis. I greatly appreciate your change to using individual values in the statistical analysis; however, I remain mildly confused by the statistical approaches taken in the paper. Most of the analyses would seem to be appropriate for two-way ANOVAs looking at genotype (YAP KO vs. Control) and/or genotype x treatment interactions (KO vs. control x TGF-b), rather than a simple one-way ANOVA. I guess the current approach is not incorrect, but the ANOVA result is not very interesting and the readers need to look at the post-tests for useful information. The use of Fisher's LSD tests for post-tests is OK for exploratory data when labeled clearly as the authors have done; it is of course less rigorous than any correction for multiple testing. I think the argument regarding using log scale vs. fold expression is not settled in the literature; while CT values would not be expected to be normally distributed, they are the directly measured variable. Other authors (such as Yuan et al., BMC Bioinformatics 2006 PMID 16504059) suggest performing the statistical analysis using the measured variable. Given that this appears to be an area of continued controversy, I withdraw my objection. The authors should include their qPCR analysis description as part of their methods (at present it must be extrapolated from CHIP-qPCR). Finally, it is appreciated that data that failed normalization tests were analyzed with non-parametric methods; lack of failure is not a strong guarantee of normality with such small numbers, but (in my opinion) attempted reanalysis at this point would require additional replicates and be prohibitively time-intensive.
As stated by the reviewer, since there is still a bit of controversy in the literature regarding the use of log scale vs. fold expression for the visualization of data and the statistical analysis, we agreed to include a detailed description of the analysis performed for the qPCR data. We have now added a specific dedicated section in the Methods paragraph at page 23.

Minor comments:
Figure S1F: I do not understand the notation on the 2-way ANOVA results. Presumably the two-way is for time and genotype. The ANOVA should be statistically significant for time, possibly genotype and definitely time x genotype interaction, and I would report all 3 values. It would seem the relevant post-hoc comparisons would be between the 2 groups at the same timepoint, not to t=0.
We apologies with the reviewer for not being enough clear. The ANOVA was indeed statistically significant for time and genotype, and the post-hoc comparison performed was between the 2 genotypes at the same time point. For sake of clarity, we have now corrected the text of the legend of the figure.

© 2021. Published by The Company of Biologists under the terms of the Creative Commons Attribution License (https://creativecommons.org/licenses/by/4.0/).

Figure S4: I do not understand how n=3 experiments has produced n=8 datapoints. I think this is fairly weak evidence for an effect in primary cells. A sentence or two on the limitations of immortalized lines and why they were advantageous for use in this study (along the lines that have been argued in the rebuttal) would strengthen the paper.
We thank the reviewer for this comment that allowed us to clarify the experimental set up. For this set of experiments, ECs isolated from each brain were seeded on a single well of a 12-well plate. We have analyzed 8 wells (isolated from 8 different brains) per condition divided in three experiments performed independently. Each data point of the graph represents a well, therefore a biological replicate. We have changed the text of the figure legend to: "…n=8 biological replicates".
As requested, we also added a better sentence explaining the advantages of the use of cell lines in this research in the Results section at pag 7, and we added also proper references about this crucial aspecthuman

Page 20: The discussion text states that "LiCl treatment was capable of increasing total SMAD3 protein level accumulation along with EndMT gene expression in TGFB-stimulated YAP KO cells." LiCl wasn't able to increase EndMT gene expression in KO cells - in fact, it is interesting that YAP appears to increase Smad3 levels and nuclear localization in a pS204 / GSK3B-dependent manner, but that reversal of this is insufficient to allow transcription of these TGF-beta-dependent / EndMT genes, indicating the need for synergistic transcriptional activity. This has been commented on earlier in the discussion (page 19); however I would suggest changing this part of the discussion to follow the paper - first findings about SMAD3 protein levels, next findings about sMAD3 turnover, and last findings about transcriptional synergy. This would require moving the end sentences of the large paragraph on page 19 to page 20.
We thank the reviewer and following this advice we reshaped accordingly the Discussion section at pag 20.

Reviewer 3 Advance Summary and Potential Significance to Field:The authors have addressed my concerns except the validation of their major findings in primary human endothelial cells. See my comments below:

Reviewer 3 Comments for the Author:
The author claim that because of the presence of TGFb in the serum of the culture media, serum starvation is required in order to achieve an appreciable TGFb stimulation. While TGFb is present in serum (very low concentration), it is usually in its latent form or is bound to blood proteins such as macroglobulin and then is inactive. Also, the majority of commercially available human endothelial cell culture media contain 5% FBS or less, further reducing the presence of active TGFb. Because the concentration of TGFb in serum is significantly lower than the recombinant one (5ng/ml) that was used to treat cells, the exogenous TGFb should activate this signaling even in presence of 5% serum. Alternatively, to reduce any potential contamination from active TGFb the author should reduce the serum concentration to 0.5-1% which should maintain endothelial monolayer for at least 18-24 hours (which is a sufficient time for TGFb stimulation). In addition there are numerous papers in which EndoMT was obtained in vitro in TGFb-treated HUVECs and other primary human endothelial cells. Here is one example (PMID: 17194892).

We thank the reviewer for the suggestion. We would like to clarify that while performing the first round of revision, working with HUVEC as a primary model system, all the following conditions were already tested:

- *0% serum starvation*
- *1% - 2% and 5% low serum starvation*
- *1% BSA starvation*

and we treated with increasing doses of TGF-β from 1 up to 100ng/ml.

In all the tested conditions the junctions are rapidly dismantled and cell retraction occurs. This phenomenon inhibited the proper response to TGF-β1 (Rudini et al., 2008) and we were not able to see any correct activation of the signal in terms of upregulation of target genes, as reported below:

© 2021. Published by The Company of Biologists under the terms of the Creative Commons Attribution License (https://creativecommons.org/licenses/by/4.0/).

Experiment 1:

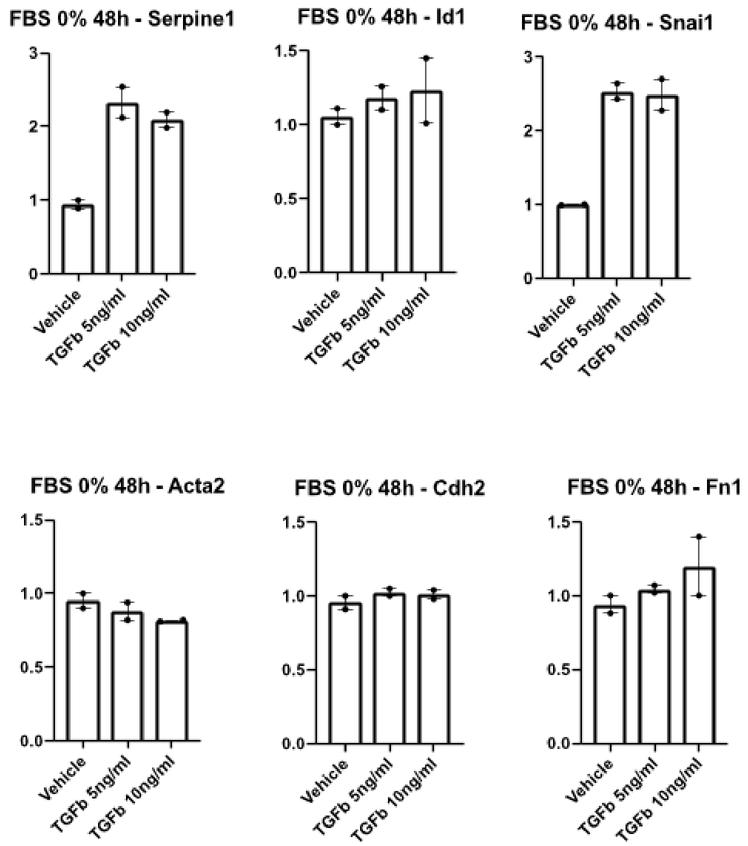

HUVEC cells cultured in MCDB131+FBS 20%, starved in MCDB131 without FBS overnight and

© 2021. Published by The Company of Biologists under the terms of the Creative Commons Attribution License (https://creativecommons.org/licenses/by/4.0/).

stimulated with TGF-β1 5-10ng/ml for 48 hours. Cells slightly responded to TGF-β1 but the monolayers were not intact.

Experiment 2:

© 2021. Published by The Company of Biologists under the terms of the Creative Commons Attribution License (https://creativecommons.org/licenses/by/4.0/).

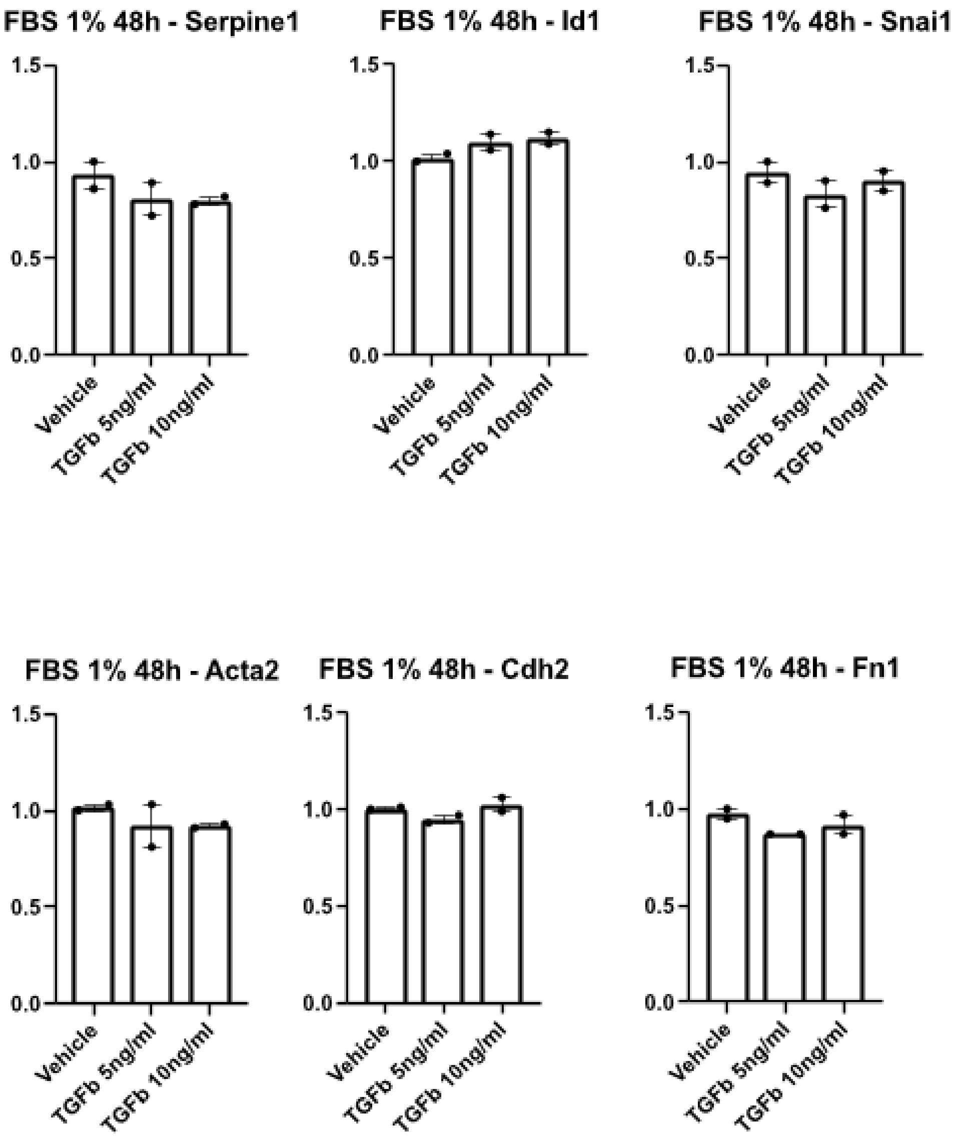

HUVEC cells cultured in MCDB131+FBS 20%, starved in MCDB131 with FBS 1% overnight and stimulated with TGF-β1 5-10ng/ml for 48 hours. Cells were suffering with disrupted monolayer and did not respond to TGF-β1.

Experiment 3:

© 2021. Published by The Company of Biologists under the terms of the Creative Commons Attribution License (https://creativecommons.org/licenses/by/4.0/).

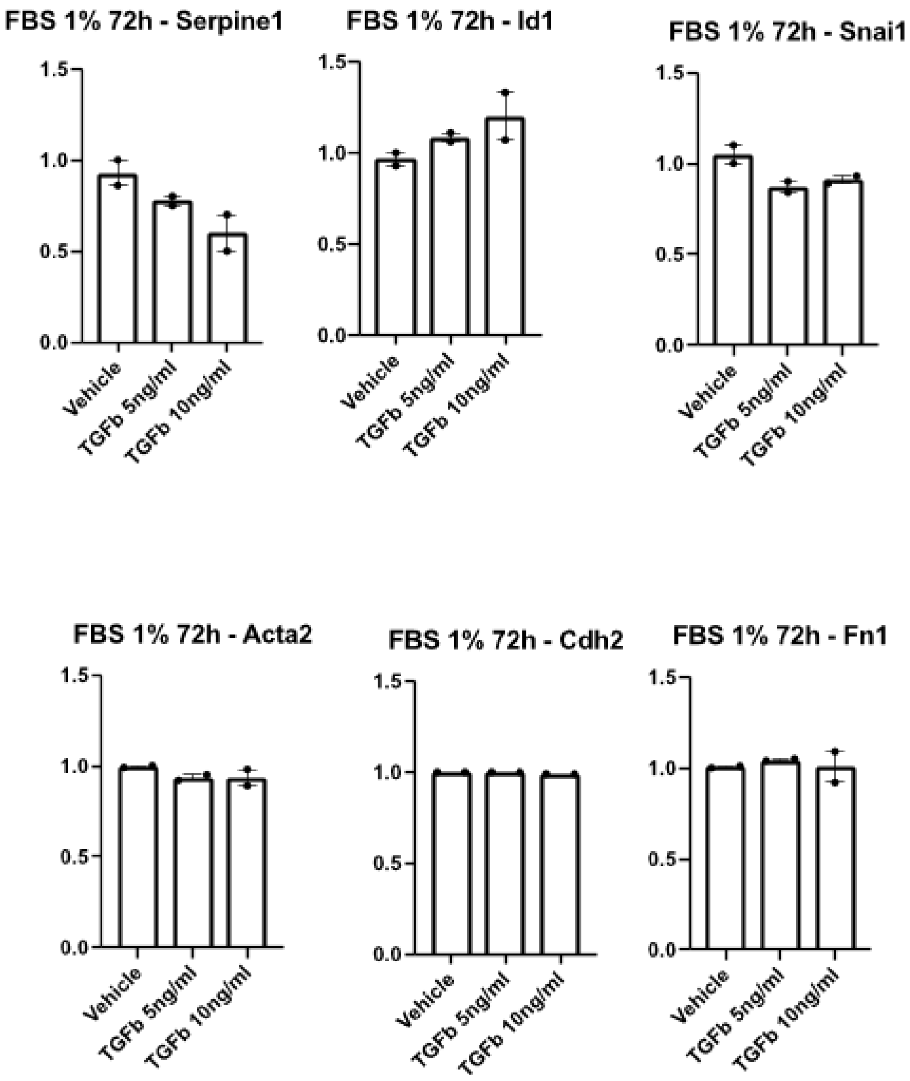

HUVEC cells cultured in MCDB131+FBS 20%, starved in MCDB131 with FBS 1% overnight and stimulated with TGF-β1 5-10ng/ml for 72 hours. Cells were suffering with disrupted monolayer and did not respond to TGF-β1.

Experiment 4:

© 2021. Published by The Company of Biologists under the terms of the Creative Commons Attribution License (https://creativecommons.org/licenses/by/4.0/).

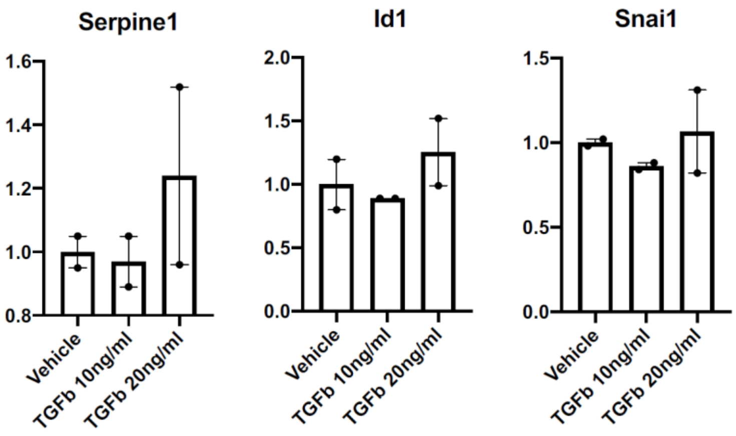

HUVEC cells cultured in MCDB131+FBS 20%, starved in MCDB131 with FBS 2% overnight and stimulated with TGF-β1 10-20ng/ml for 48 hours. Cells looked better with good monolayer, but did not respond to TGF- β1.

Experiment 5:

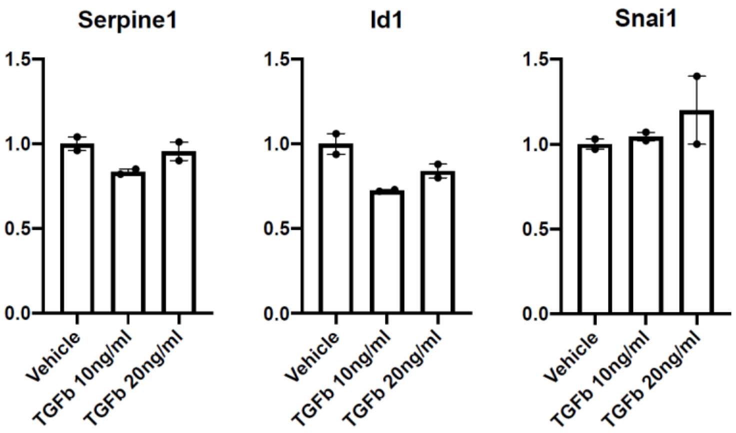

HUVEC cells cultured in MCDB131+FBS 20%, starved in MCDB131 with FBS 5% overnight and stimulated with TGF-β1 10-20ng/ml for 48 hours. Cells looked good with good monolayer, but did not respond to TGF-β1.

Experiment 6:

© 2021. Published by The Company of Biologists under the terms of the Creative Commons Attribution License (https://creativecommons.org/licenses/by/4.0/).

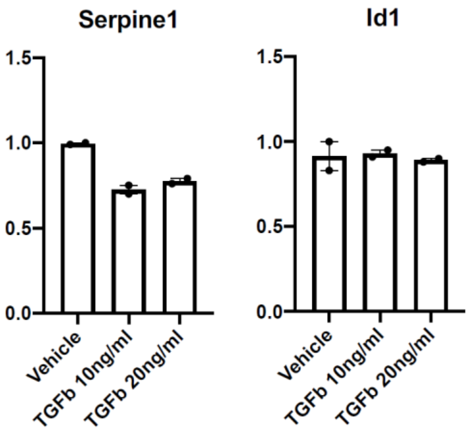

HUVEC cells cultured in MCDB131+FBS 5%, starved in MCDB131 with BSA 1%+ITS for 2 hours and stimulated with TGF-B1 10-20ng/ml for 24 hours. Cells were not good and did not respond to TGF-B1.

Experiment 7:

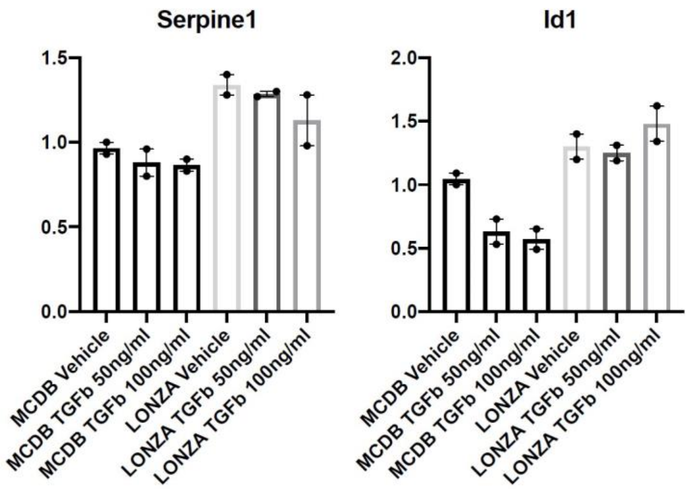

HUVEC cells cultured in either MCDB131 or LONZA +FBS 5%, starved in either MCDB131 or LONZA with BSA 1% overnight and stimulated with TGF-B1 50-100ng/ml for 24 hours. Cells were good but did not respond to TGF-B1. Slight difference between media.

Experiment 8:

© 2021. Published by The Company of Biologists under the terms of the Creative Commons Attribution License (https://creativecommons.org/licenses/by/4.0/).

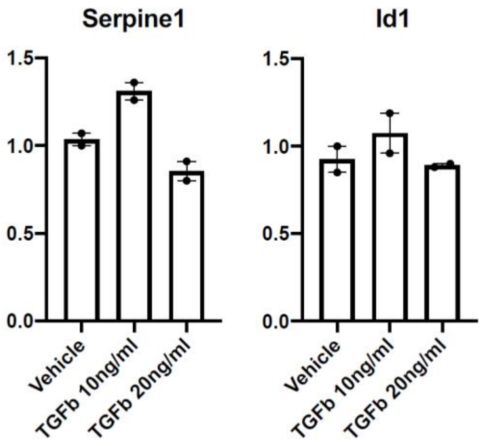

HUVEC cells cultured in LONZA +FBS 20%, starved in either LONZA with BSA 1% overnight and stimulated with TGF-β1 10-20ng/ml for 24 hours. Cells were good but did not respond to TGF-β1

Experiment 9:

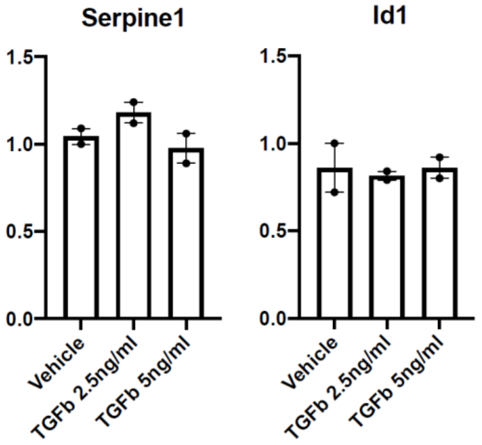

HUVEC cells cultured in LONZA +FBS 20%, starved in LONZA with BSA 1% overnight and stimulated with TGF-β1 2.5-5ng/ml for 48 hours. Cells were good but did not respond to TGF-β1.

Experiment 10:

© 2021. Published by The Company of Biologists under the terms of the Creative Commons Attribution License (https://creativecommons.org/licenses/by/4.0/).

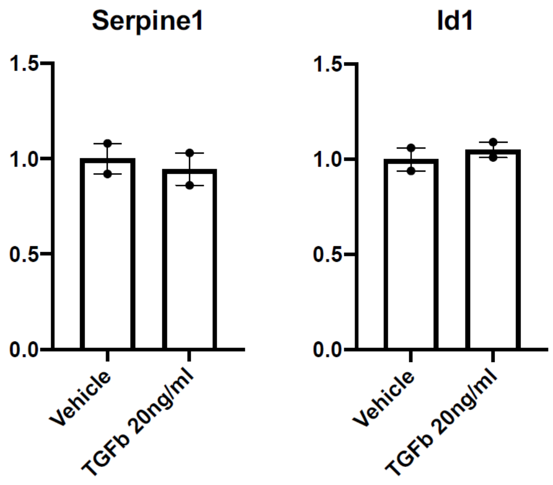

HPMEC cells cultured in MCDB131 +FBS 10%, starved in MCDB131 + BSA 1% + FBS 0.5% for 5 hours and stimulated with TGF-β1 20ng/ml for 24 hours. Cells were suffering and did not respond to TGF-β1.

Experiment 11:

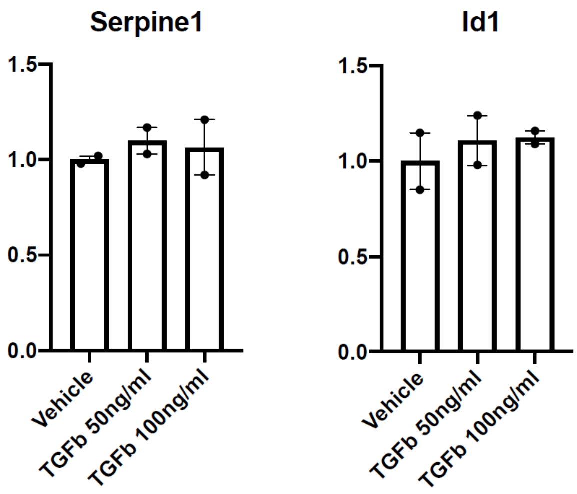

HPMEC cells cultured in EC Growth Medium MV from Promocell starved in the same medium without constituents + 1% BSA overnight and stimulated with TGF-β1 50-100ng/ml for 24 hours. Cells were good, but did not respond to TGF-β1.

Experiment 12:

© 2021. Published by The Company of Biologists under the terms of the Creative Commons Attribution License (https://creativecommons.org/licenses/by/4.0/).

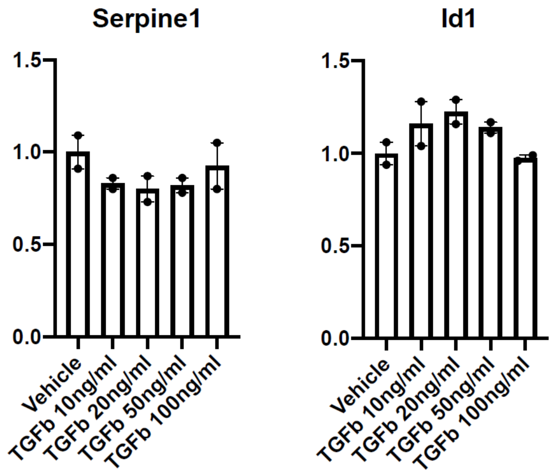

HPMEC cells cultured in EC Growth Medium MV from Promocell starved in the same medium without constituents + 1% BSA overnight and stimulated with TGF-β1 10-20-50-100ng/ml for 24 hours. Cells were good but did not respond to TGF-β1.

Experiment 13:

Moreover, to test the effect of TGF-β present in the FBS, we did the following experiment:

1) HPMEC cells cultured in EC Growth Medium MV from Promocell starved in the same medium without constituents + 1% BSA for 8 hours and stimulated with TGF-β1-5ng/ml for 45 minutes.

2) HPMEC cells cultured in EC Growth Medium MV from Promocell and stimulated with TGF-β1 5ng/ml for 45 minutes without serum starvation.

We then extracted the protein and run WB for total SMAD3 (right part of the blot) and pSMAD3 (left part of the blot).

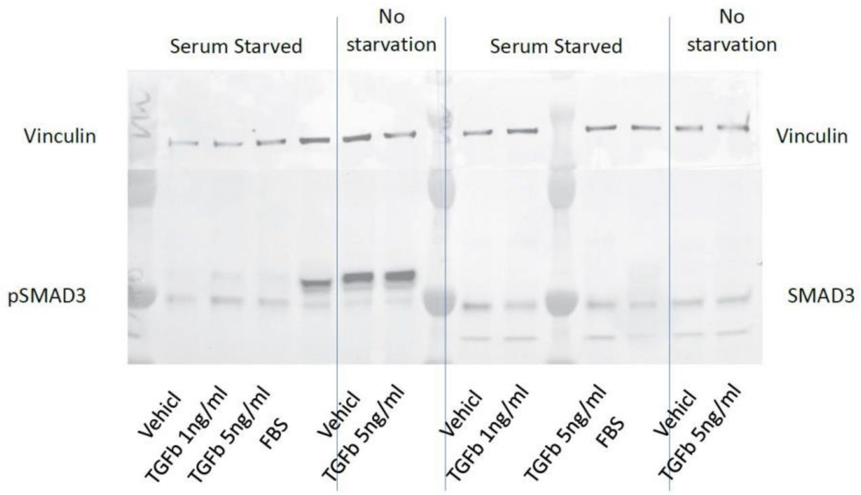

The results clearly showed that the TGF-β present in the FBS is indeed active and induces

© 2021. Published by The Company of Biologists under the terms of the Creative Commons Attribution License (https://creativecommons.org/licenses/by/4.0/).

strong phosphorylation of SDMA3, much stronger than the exogenous TGF-β1 at 5ng/ml. To be noted that EC Growth Medium MV from Promocell contains only 2% of FBS, which is however still very active on SMAD3 pathway. Under these conditions, the TGF-β/SMAD3 pathway is constitutively activated thus cells do not further respond to exogenous TGF-β1 stimuli. Moreover, this chronic stimulation may require very longconnections and good monolayer integrity.

Finally, looking thorough the literature as suggested, we found out that TGF-β pathway activation (shown only as SMAD phosphorylation, which cannot be considered as EndMT induction) can be obtained in HUVEC cells although the integrity of cell contacts is not checked or shown (Boon et al., 2007), and sometimes the subconfluent conditions of the cells is reported (Zhang et al., 2014). Moreover, in the specific paper suggested by the reviewer, TGF β3 and not TGF β1 stimulation was performed on HUVEC cells and only SMAD2 phosphorylation was used as readout.

Given the knowledge that:

1- *the different TGF-β isoforms are each required in vivo as the isoform-specific null mice are lethal (Memon et al., 2009)*
2- *the TGF-β1 - β2 and β3 null mice have different phenotypes (Memon et al., 2009; Sanford et al., 1997; Voisin et al., 2020)*
3- *in other model systems (wound skin healing) TGF-β1 and TGF-β3 have opposing biological effects (Stefan et al., 1996; Penn et al., 2012; Pakyari et al., 2013)*

we cannot guarantee that the stimulation with a different ligand would act through YAP in modulation cell responses.

For all these reasons, we decided to use endothelial cells isolated from mice, which are more resistant than the human counterparts, and we used them as primary model system where we were able to induce TGF- β1 responses in terms of induction of EndMT genes without compromising the monolayer integrity, recapitulating the data obtained with the immortalized cell model system.

Third decision letter

MS ID#: JOCES/2020/251371

MS TITLE: A Dual Role of YAP in Driving TGFβ-mediated EndMT

AUTHORS: Cecilia Savorani, Matteo Malinverno, Roberta Seccia, Claudio Maderna, Monica Giannotta, Linda Terreran, Eleonora Mastrapasqua, Stefano Campaner, Elisabetta Dejana, and Costanza Giampietro
ARTICLE TYPE: Research Article

I am happy to tell you that your manuscript has been accepted for publication in Journal of Cell Science, pending standard ethics checks.

© 2021. Published by The Company of Biologists under the terms of the Creative Commons Attribution License (https://creativecommons.org/licenses/by/4.0/).
